# Correction: Machine learning-based phenotypic imaging to characterise the targetable biology of *Plasmodium falciparum* male gametocytes for the development of transmission-blocking antimalarials

**DOI:** 10.1371/journal.ppat.1013814

**Published:** 2025-12-29

**Authors:** Oleksiy Tsebriy, Andrii Khomiak, Celia Miguel-Blanco, Penny C. Sparkes, Maurizio Gioli, Marco Santelli, Edgar Whitley, Francisco-Javier Gamo, Michael J. Delves

There are errors in the author affiliations. The correct affiliations are as follows:

Oleksiy Tsebriy^1^, Andrii Khomiak^2^, Celia Miguel-Blanco^3^, Penny C. Sparkes^4^, Maurizio Gioli^5^, Marco Santelli^2^, Edgar Whitley^6^, Francisco-Javier Gamo^3^, Michael J. Delves^4^

1 Ternopil Ivan Puluj National Technical University, Ternopil, Ukraine, 2 Independent researcher, Ternopil, Ukraine, 3 Global Health Medicines R&D., GlaxoSmithKline, Madrid, Spain, 4 Department of Infection Biology, London School of Hygiene and Tropical Medicine, Keppel Street, London, UK, 5 SOAS University of London, London, UK, 6 Department of Management, London School of Economics and Political Science, London, UK.
