# Supplementary material for: Machine learning-based phenotypic imaging to characterise the targetable biology of Plasmodium falciparum male gametocytes for the development of transmission-blocking antimalarials
Source: PLoS Pathog. 2023 Oct 6;19(10):e1011711. doi: 10.1371/journal.ppat.1011711 (PMC10584170; doi:10.1371/journal.ppat.1011711)
Supplement: S1 Fig — Cluster 6 represents the convergence of many clusters and consequently there was significant diversity of cell morphology within the cluster. The majority were irregular-shaped but showed ordered microtuble organisation. Bar = 3μm. (PDF) [file ppat.1011711.s004.pdf]

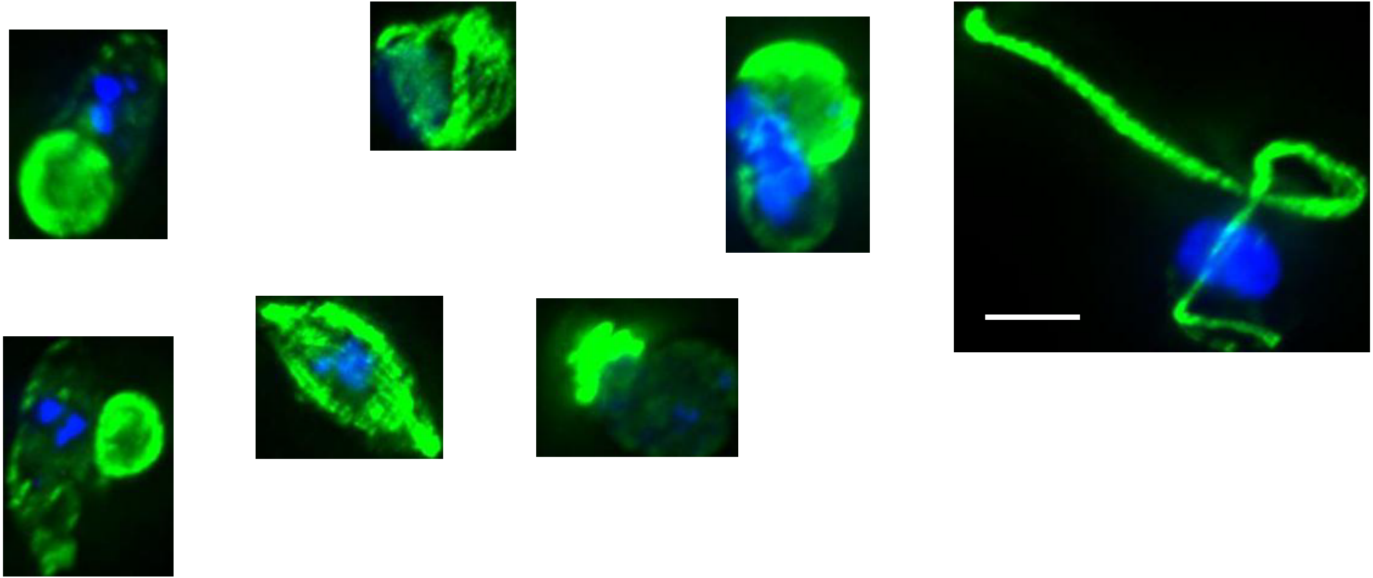

Supplementary Figure 1 - Selected cells from Cluster 6 (Fig. 4). Cluster 6 represents the convergence of many clusters and consequently there was significant diversity of cell morphology within the cluster. The majority were irregular-shaped but showed ordered microtuble organisation. Bar = 3μm.
