# Supplementary material for: Machine learning-based phenotypic imaging to characterise the targetable biology of Plasmodium falciparum male gametocytes for the development of transmission-blocking antimalarials
Source: PLoS Pathog. 2023 Oct 6;19(10):e1011711. doi: 10.1371/journal.ppat.1011711 (PMC10584170; doi:10.1371/journal.ppat.1011711)
Supplement: S2 Fig — (PDF) [file ppat.1011711.s005.pdf]

Supplementary Figure 2 – The distribution of cells from individual drug treatments visualised within the entire dataset.

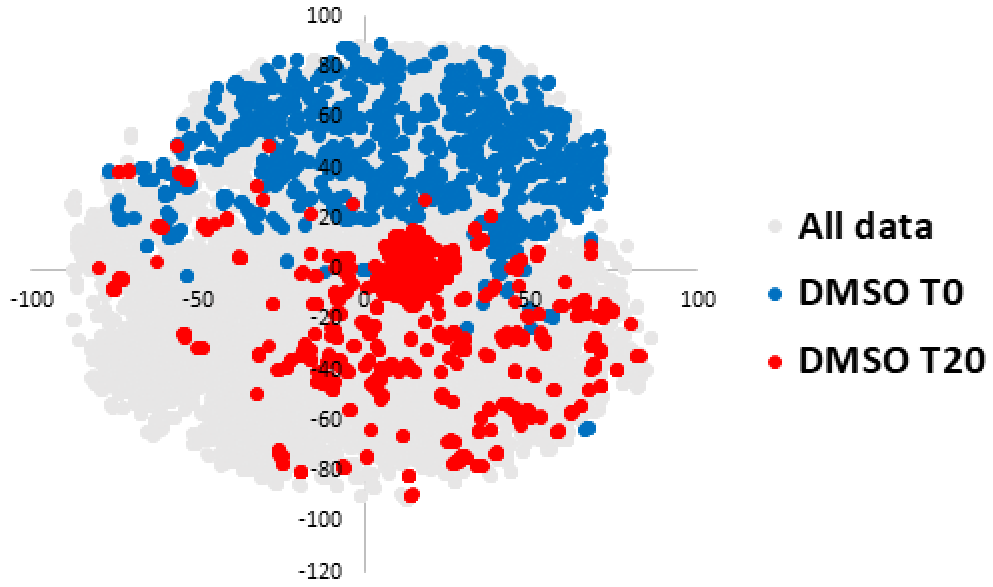
All data
DMSO T0
DMSO T20

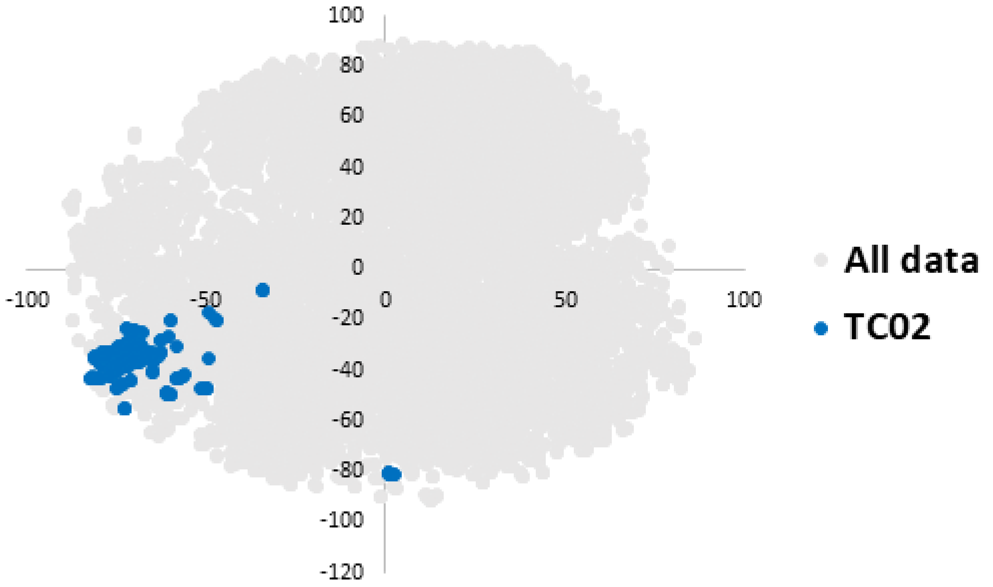
All data
TC02

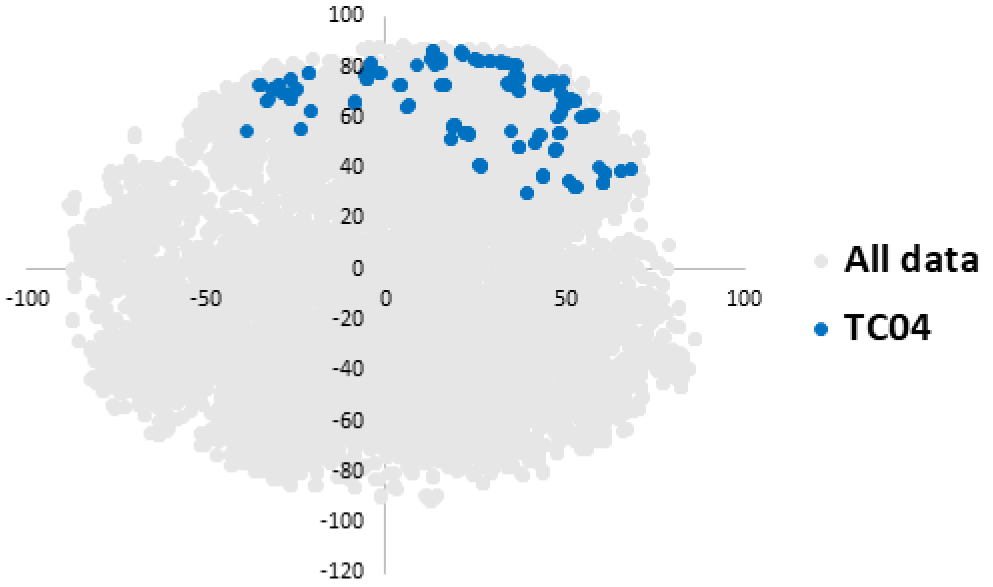
All data
TC04

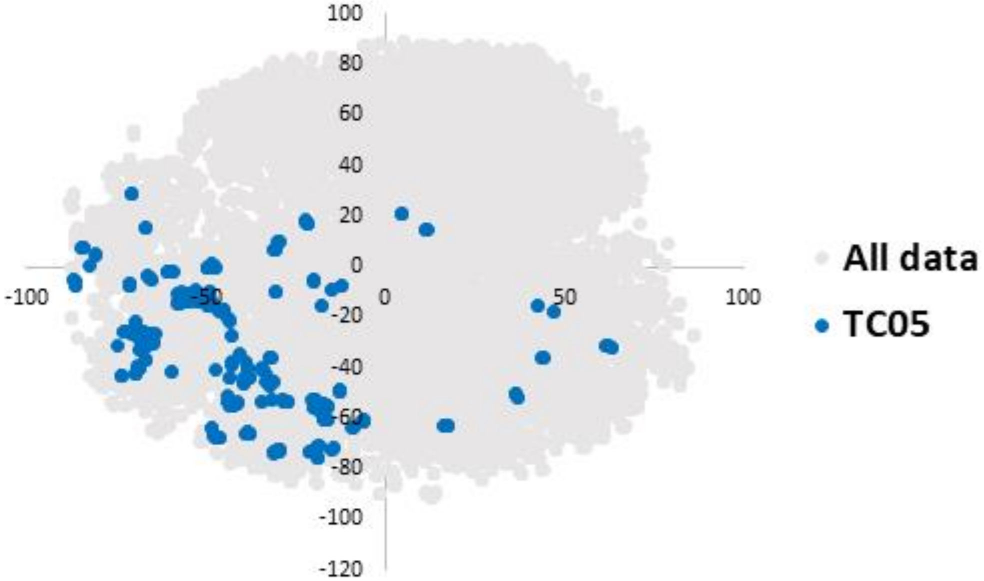
100
80
60
40
20
0
-20
-40
-60
-80
-100
-120
-100
-50
0
50
100
All data
TC05

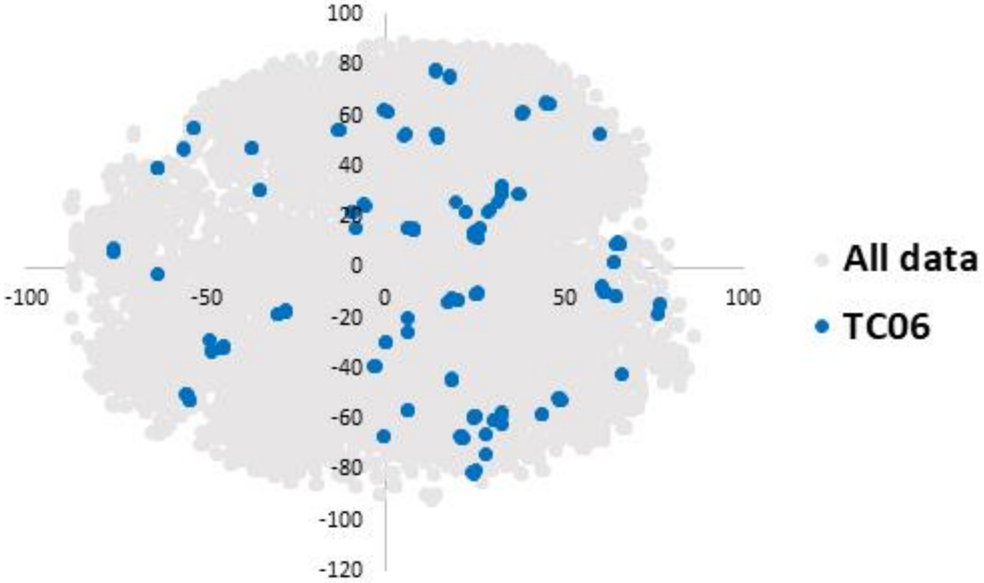
100
80
60
40
20
0
-20
-40
-60
-80
-100
-120
-100
-50
0
50
100
All data
TC06

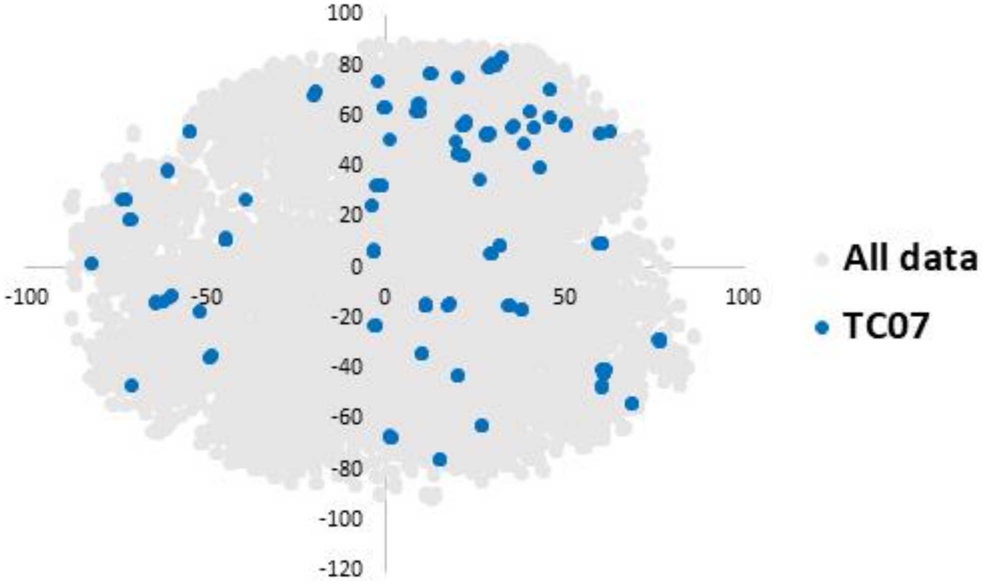
100
80
60
40
20
0
-20
-40
-60
-80
-100
-120
-100
-50
0
50
100
All data
TC07

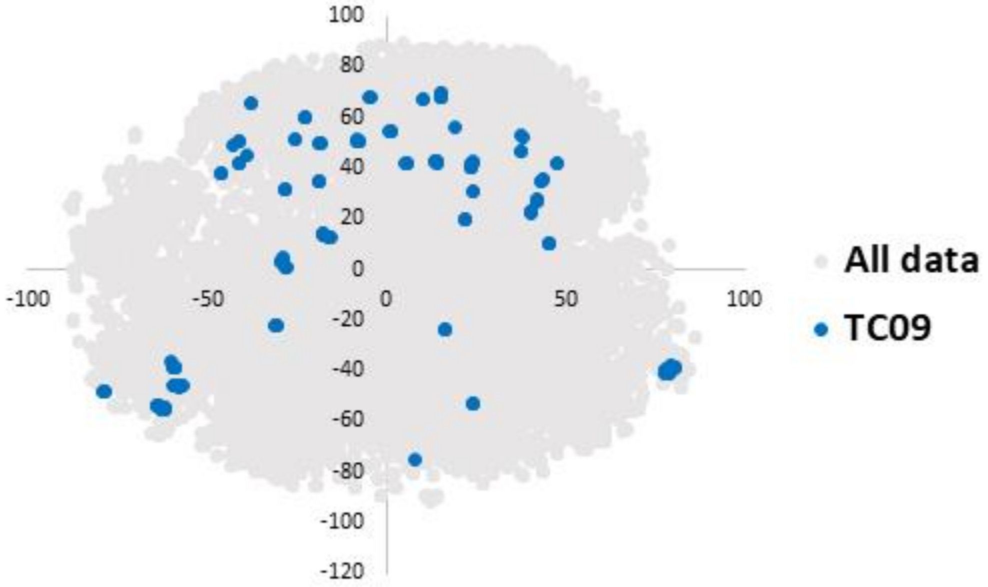
100
80
60
40
20
0
-20
-40
-60
-80
-100
-120
-100
-50
0
50
100
All data
TC09

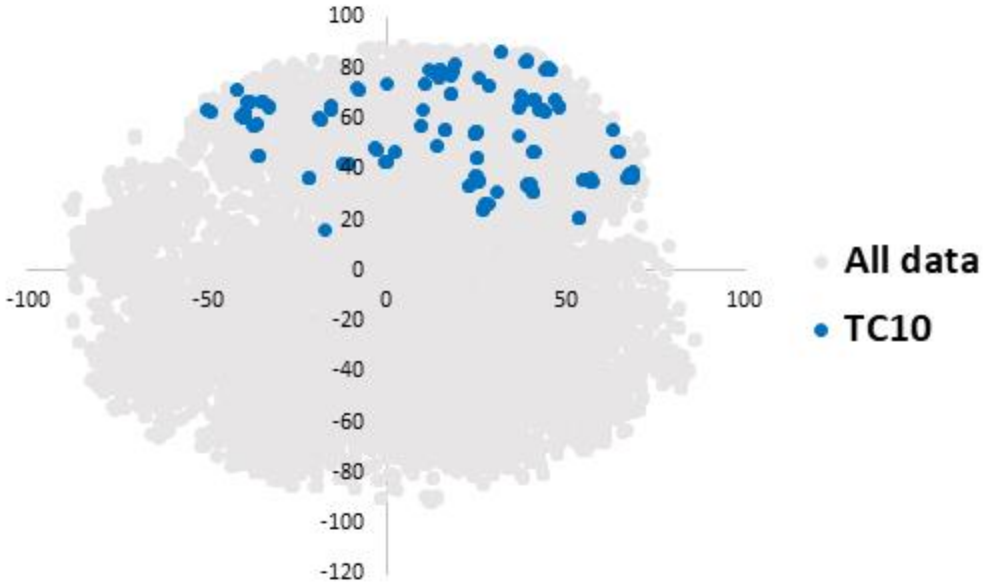
100
80
60
40
20
0
-20
-40
-60
-80
-100
-120
-100
-50
0
50
100
All data
TC10

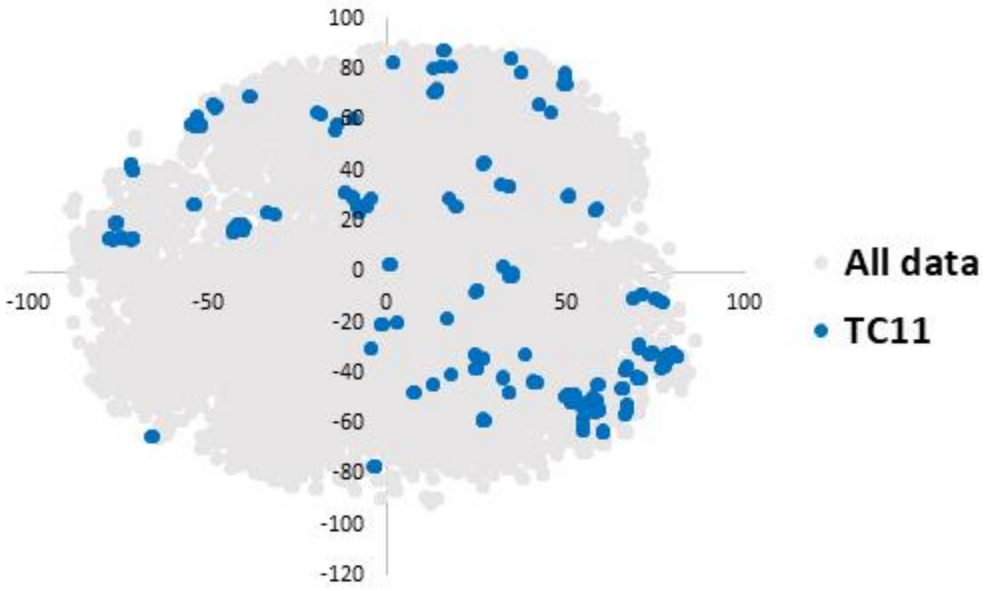
100
80
60
40
20
0
-20
-40
-60
-80
-100
-120
-100
-50
0
50
100
All data
TC11

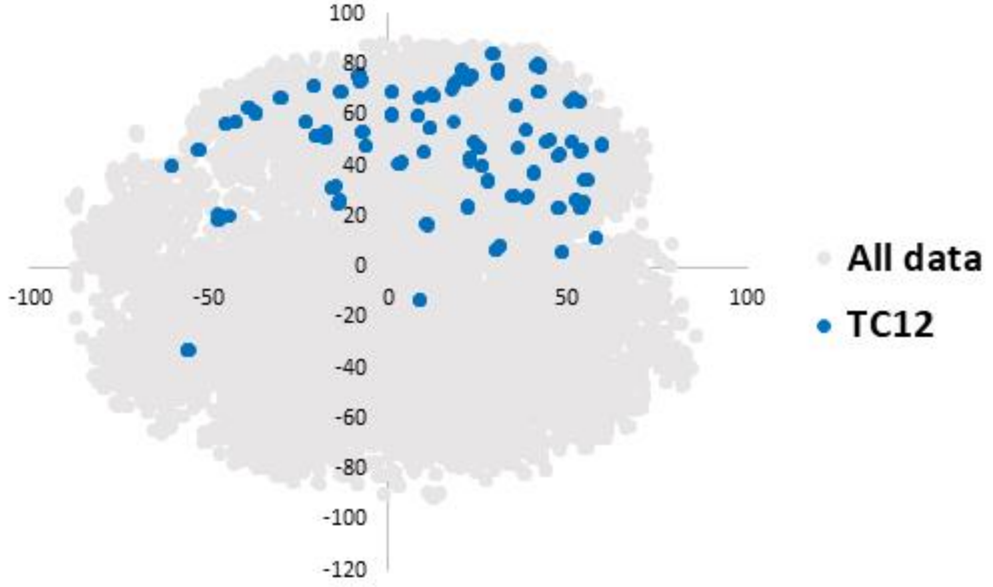
100
80
60
40
20
0
-100
-50
0
50
100
-20
-40
-60
-80
-100
-120
All data
TC12

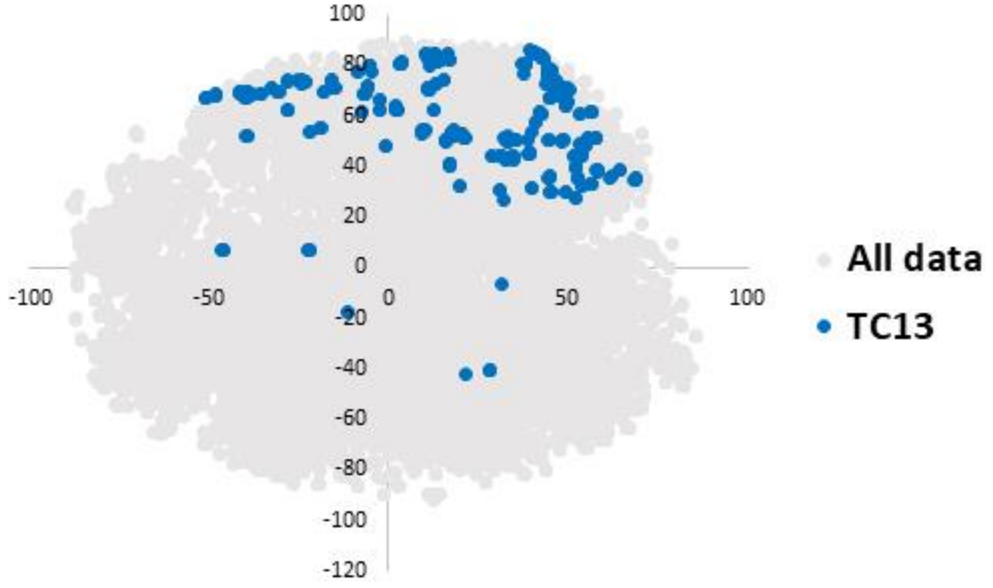
100
80
60
40
20
0
-100
-50
0
50
100
-20
-40
-60
-80
-100
-120
All data
TC13

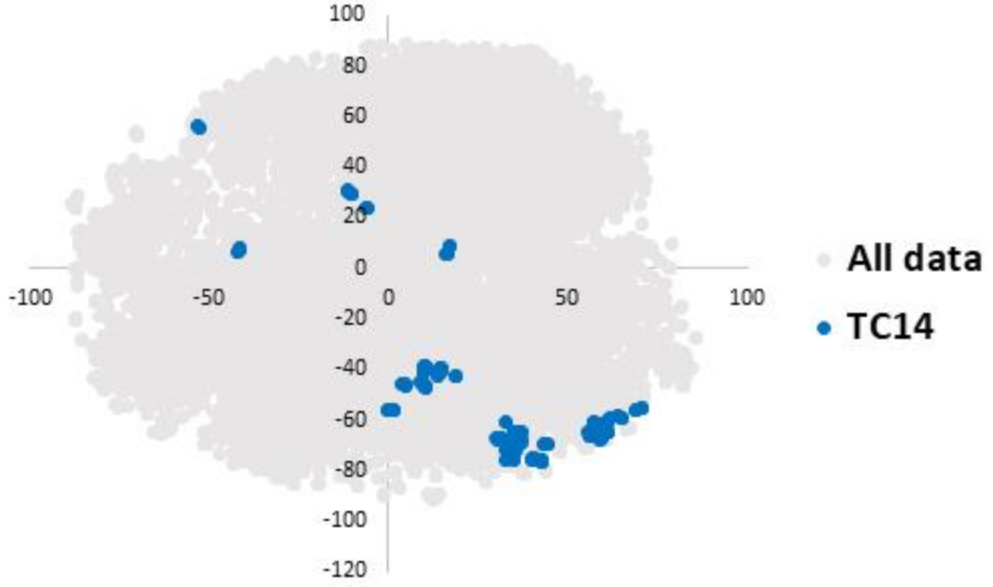
100
80
60
40
20
0
-100
-50
0
50
100
-20
-40
-60
-80
-100
-120
All data
TC14

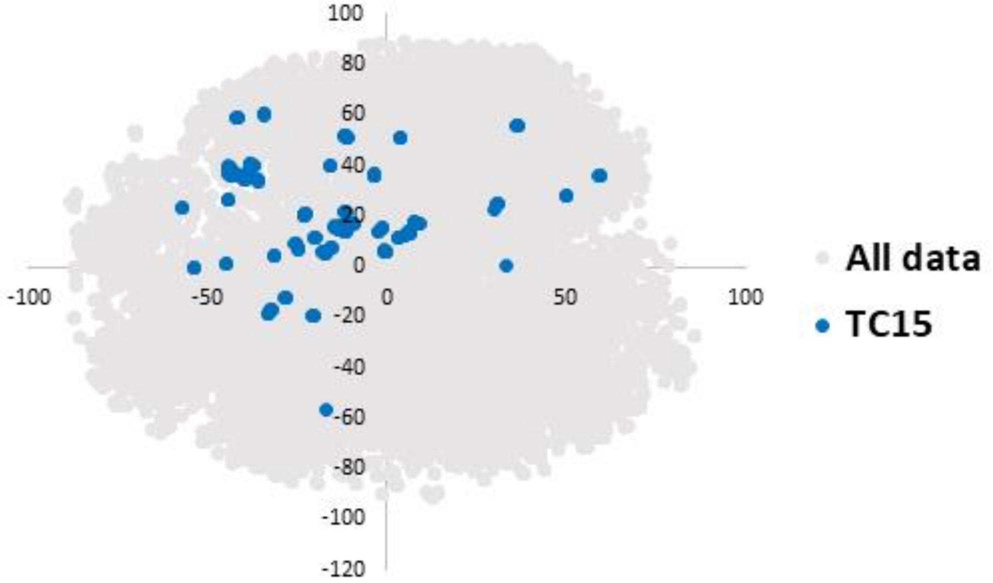
100
80
60
40
20
0
-20
-40
-60
-80
-100
-120
-100
-50
0
50
100
All data
TC15

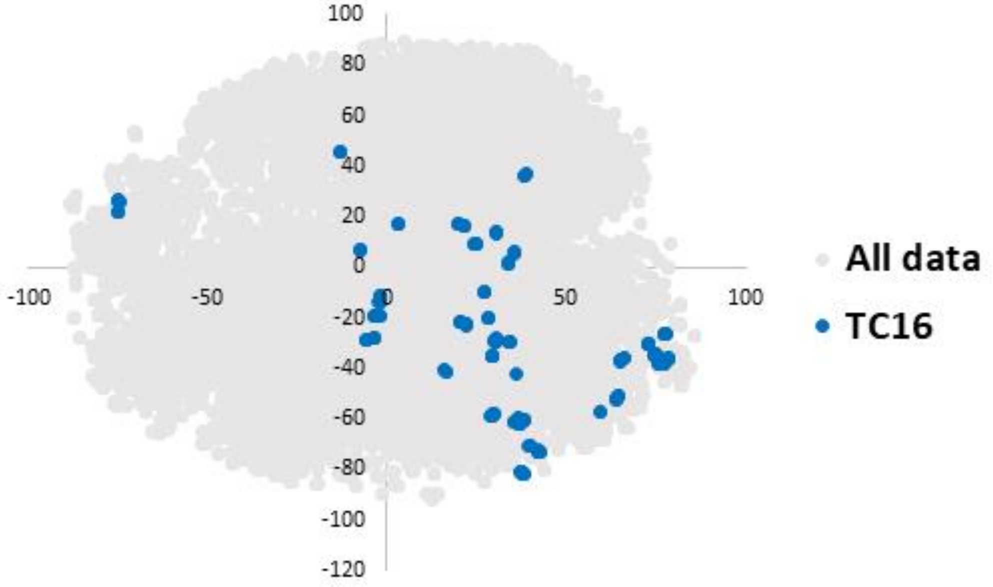
100
80
60
40
20
0
-20
-40
-60
-80
-100
-120
-100
-50
0
50
100
All data
TC16

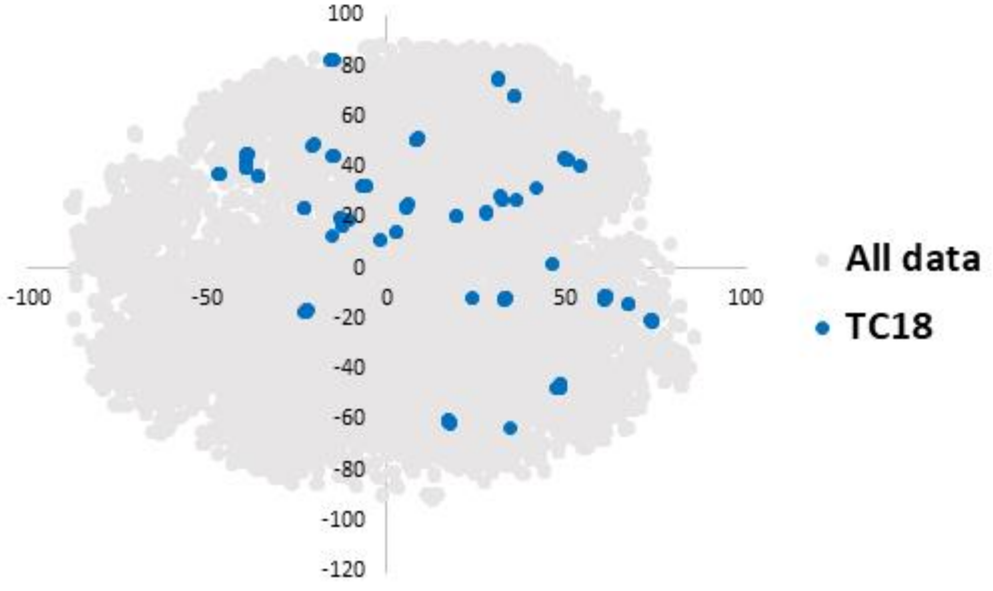
100
80
60
40
20
0
-20
-40
-60
-80
-100
-120
-100
-50
0
50
100
All data
TC18

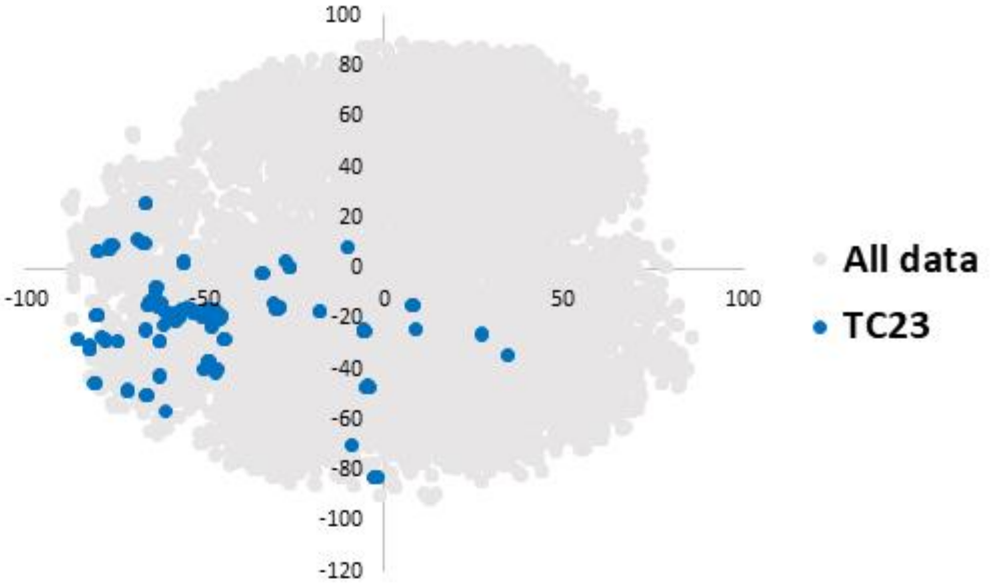

100
80
60
40
20
0
-20
-40
-60
-80
-100
-120
-100
-50
0
50
100
All data
TC23

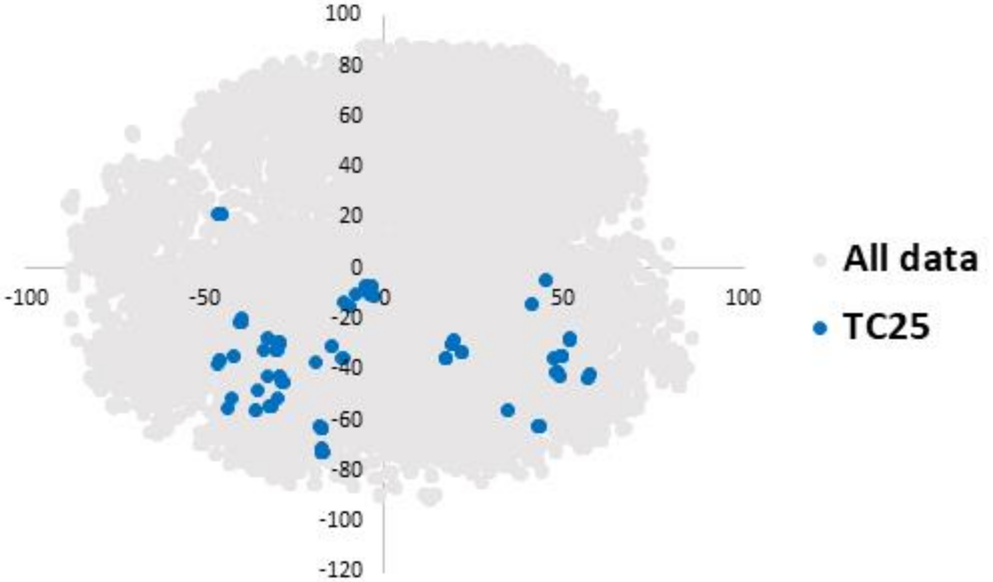

100
80
60
40
20
0
-20
-40
-60
-80
-100
-120
-100
-50
0
50
100
All data
TC25

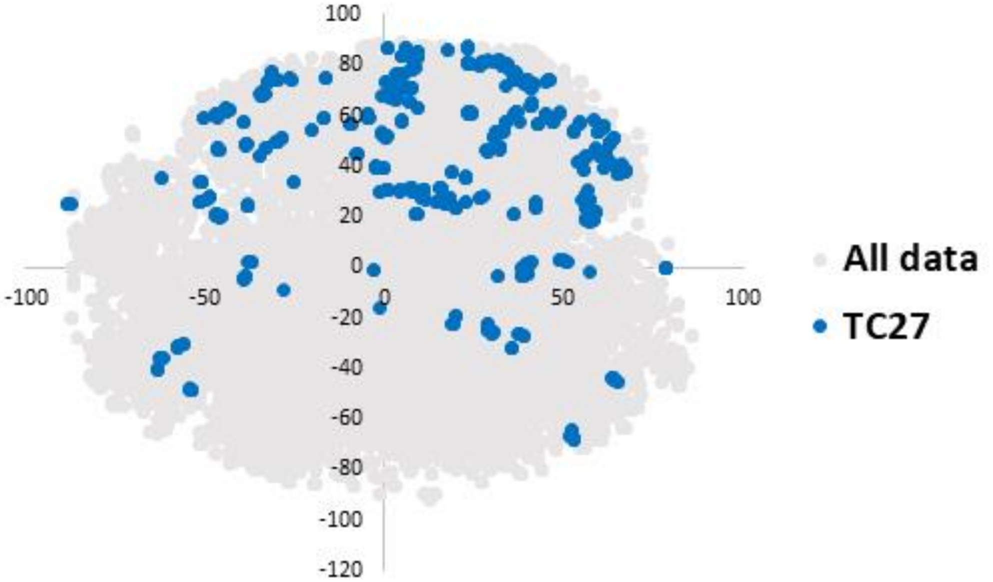

100
80
60
40
20
0
-20
-40
-60
-80
-100
-120
-100
-50
0
50
100
All data
TC27

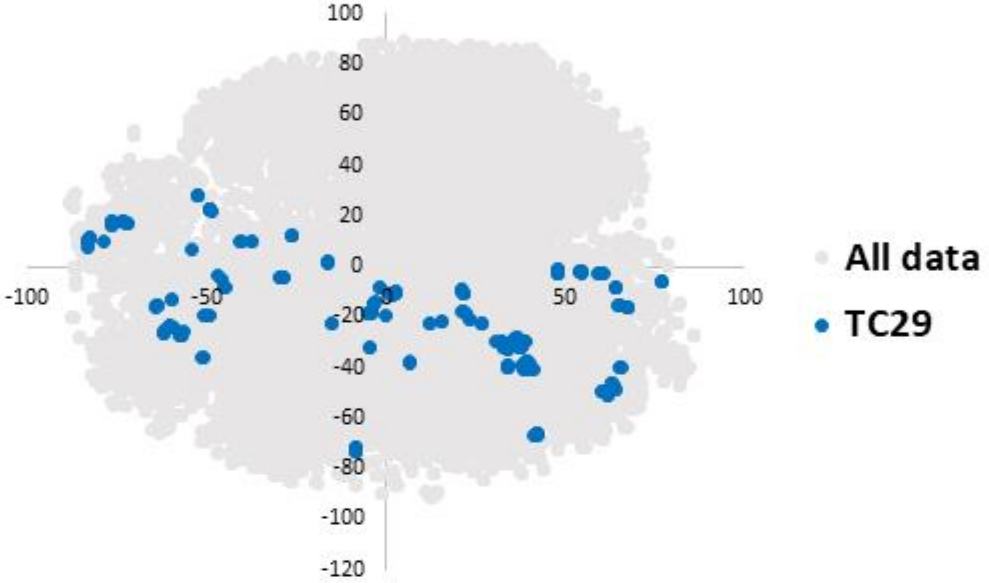
100
80
60
40
20
0
-20
-40
-60
-80
-100
-120
-100
-50
0
50
100
All data
TC29

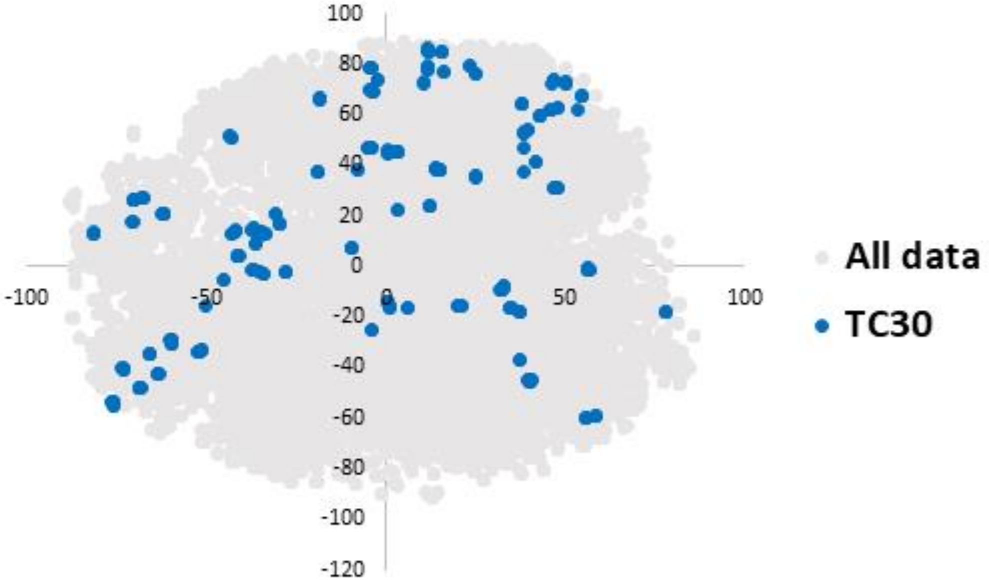
100
80
60
40
20
0
-20
-40
-60
-80
-100
-120
-100
-50
0
50
100
All data
TC30

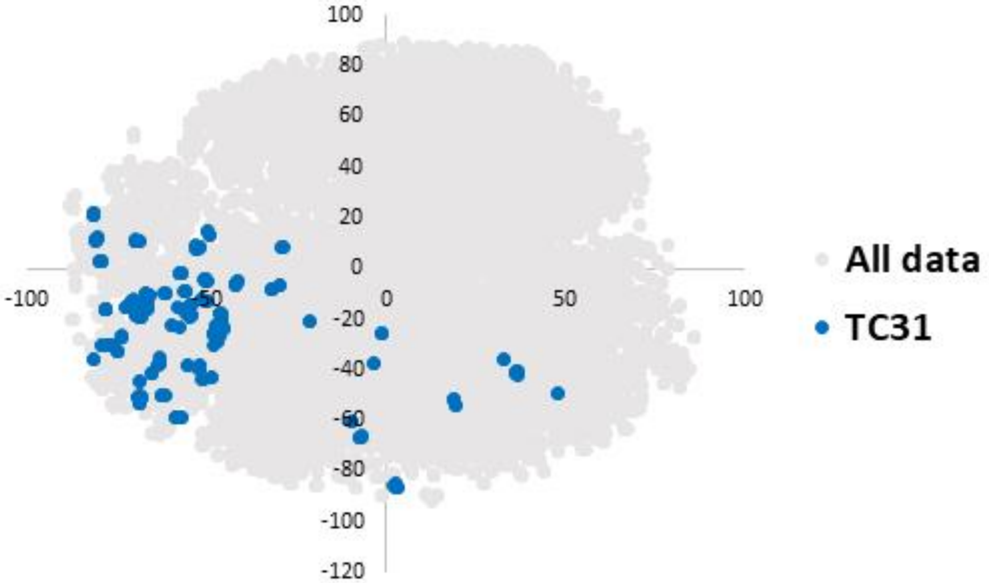
100
80
60
40
20
0
-20
-40
-60
-80
-100
-120
-100
-50
0
50
100
All data
TC31

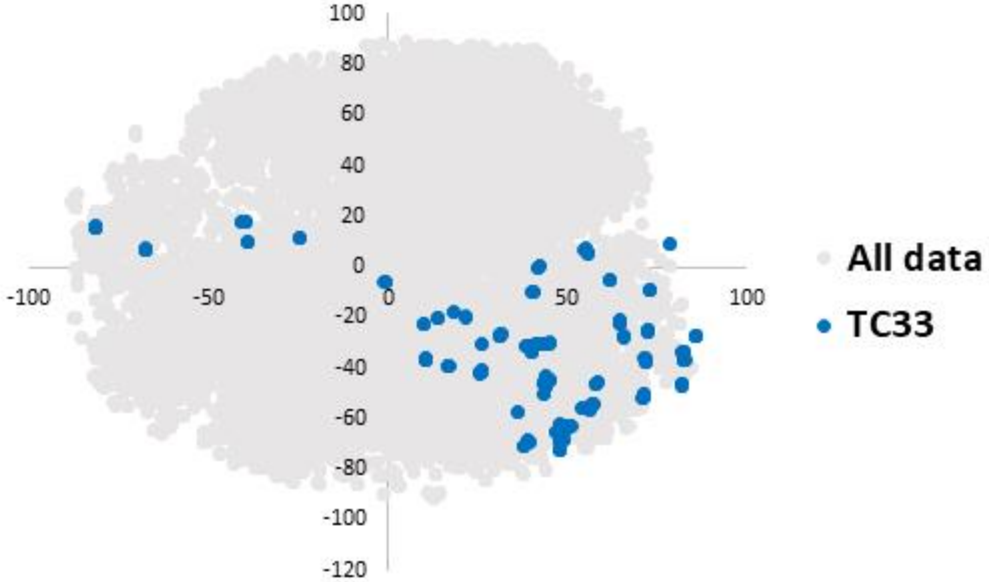

100
80
60
40
20
0
-20
-40
-60
-80
-100
-120
-100
-50
0
50
100
All data
TC33

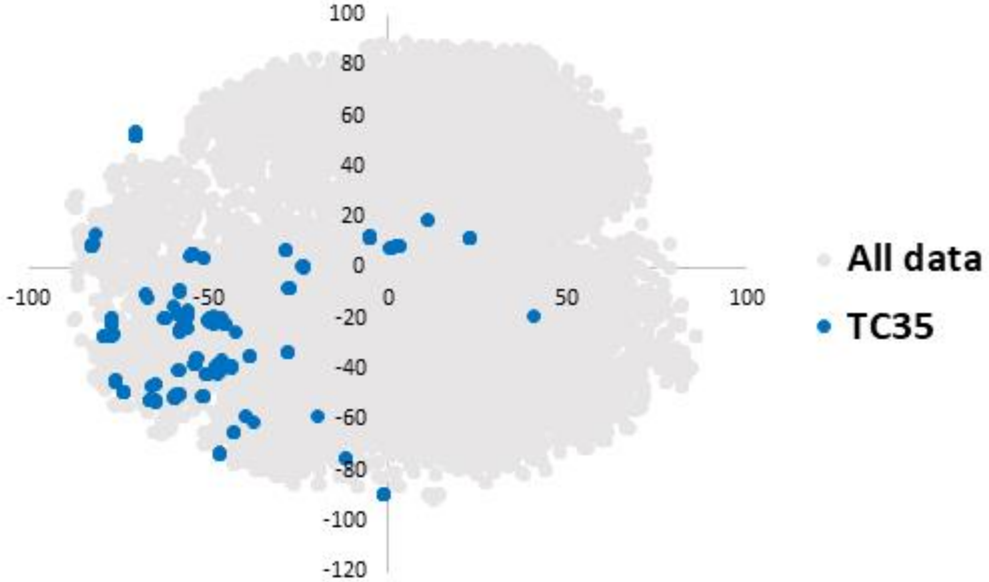

100
80
60
40
20
0
-20
-40
-60
-80
-100
-120
-100
-50
0
50
100
All data
TC35

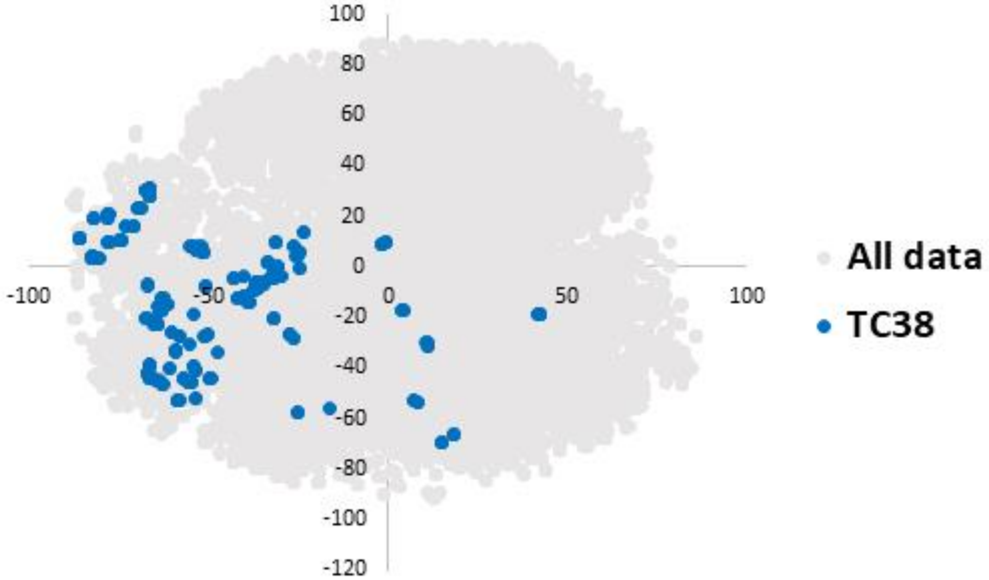

100
80
60
40
20
0
-20
-40
-60
-80
-100
-120
-100
-50
0
50
100
All data
TC38

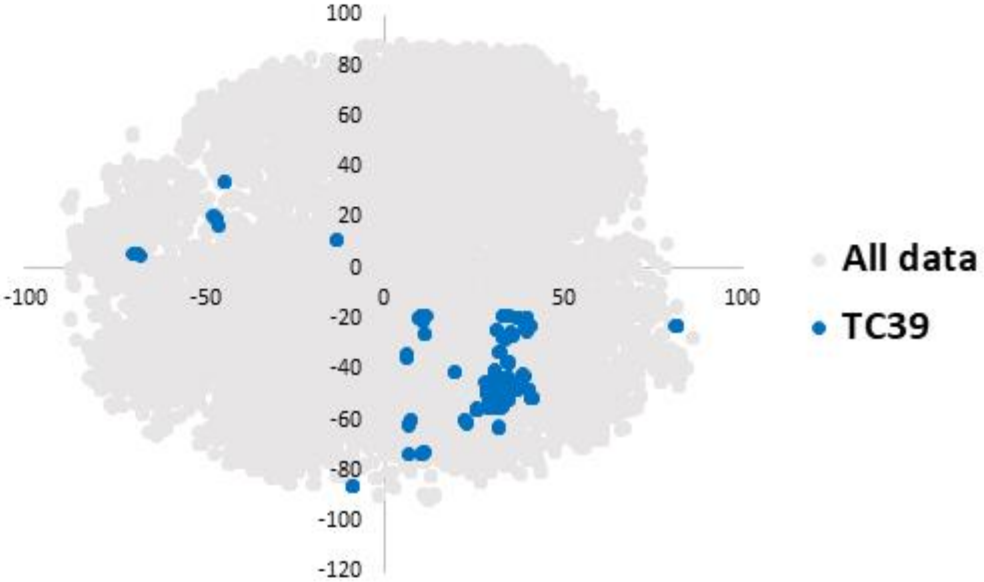
100
80
60
40
20
0
-20
-40
-60
-80
-100
-120
-100
-50
0
50
100
All data
TC39

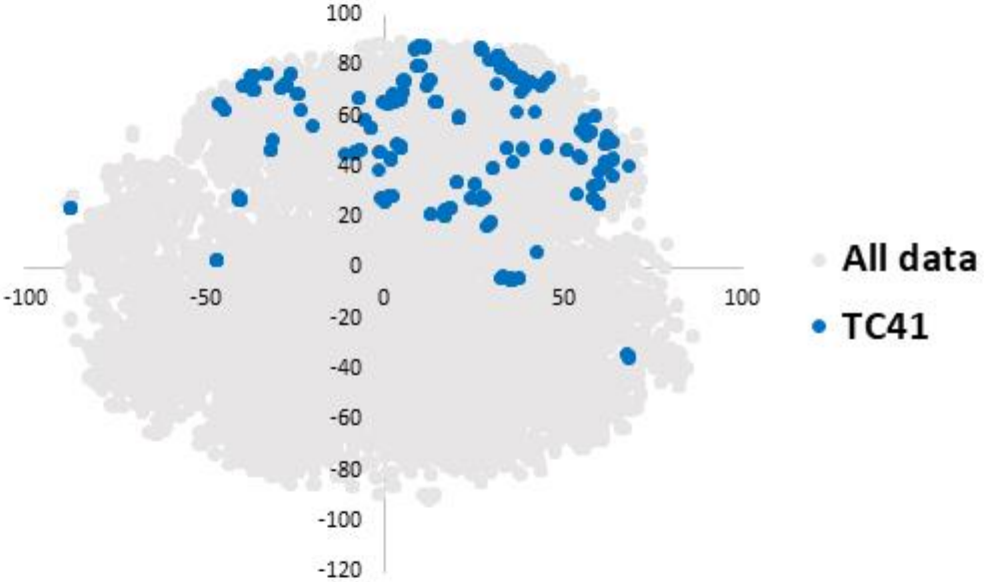
100
80
60
40
20
0
-20
-40
-60
-80
-100
-120
-100
-50
0
50
100
All data
TC41

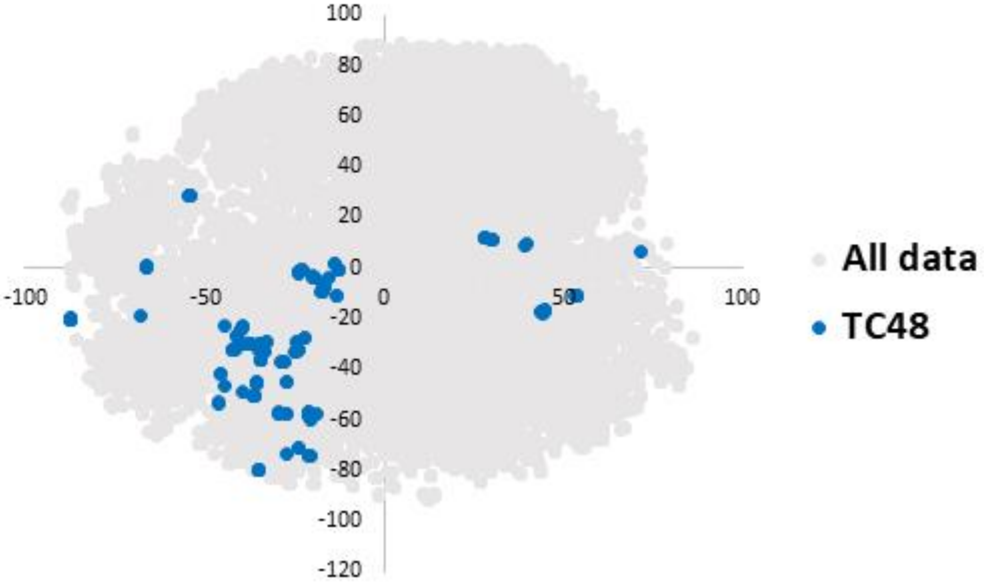
100
80
60
40
20
0
-20
-40
-60
-80
-100
-120
-100
-50
0
50
100
All data
TC48

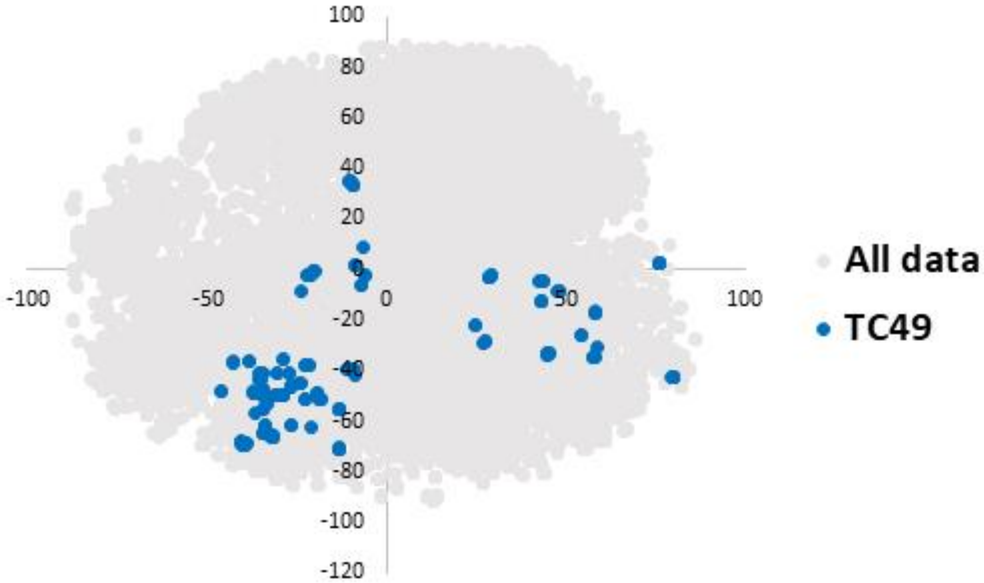
100
80
60
40
20
0
-20
-40
-60
-80
-100
-120
-100
-50
50
100
All data
TC49

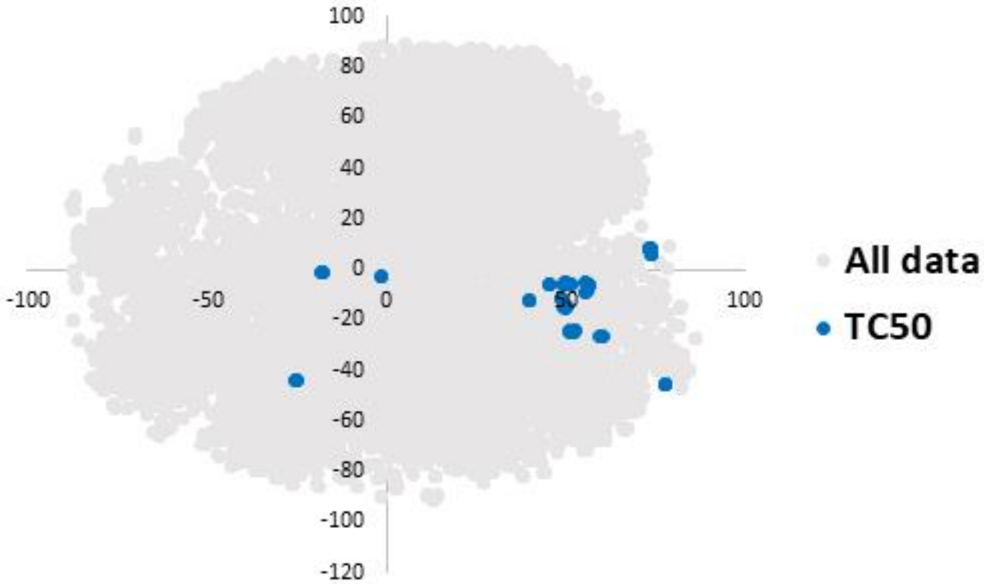
100
80
60
40
20
0
-20
-40
-60
-80
-100
-120
-100
-50
50
100
All data
TC50

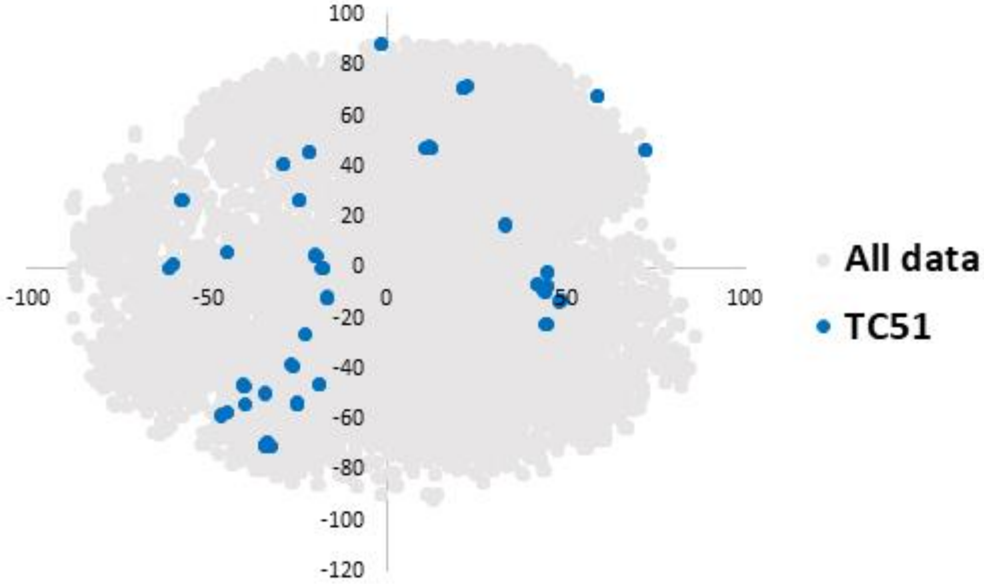
100
80
60
40
20
0
-20
-40
-60
-80
-100
-120
-100
-50
50
100
All data
TC51

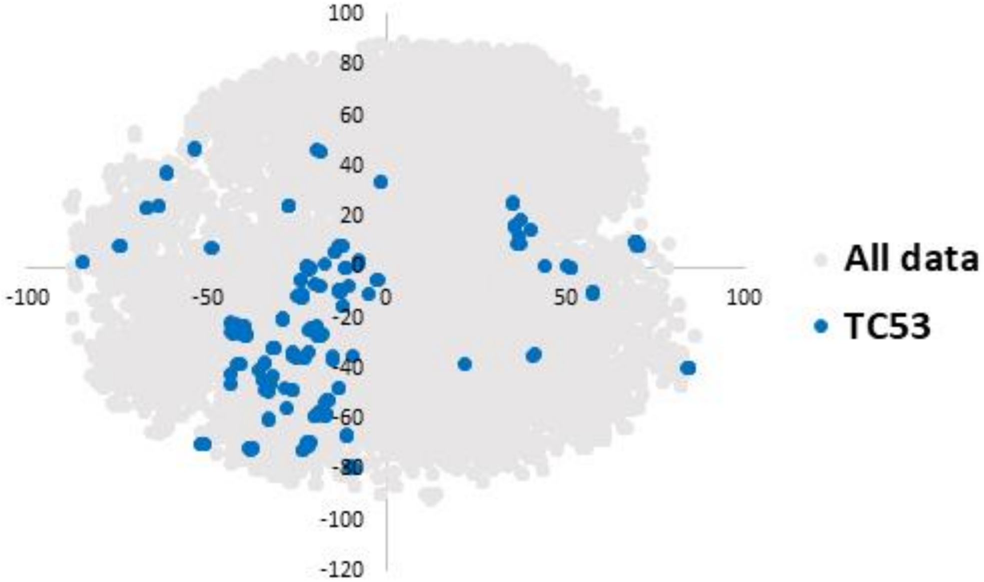

All data
TC53

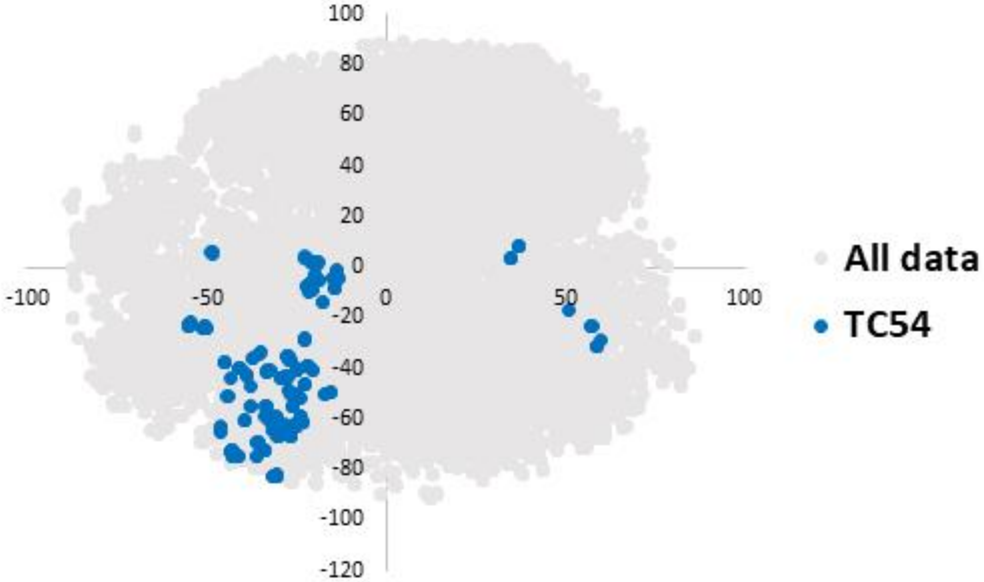

All data
TC54

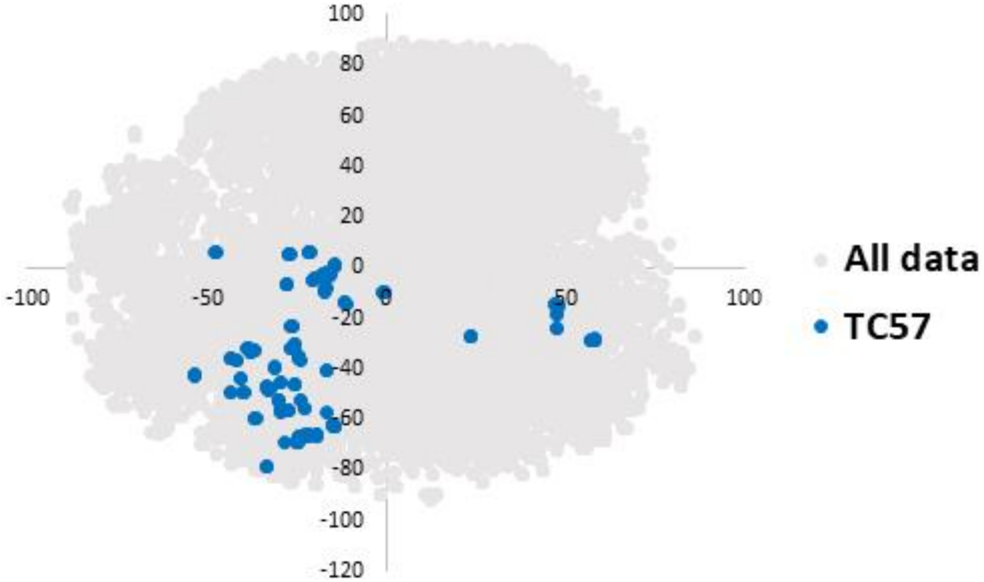

All data
TC57

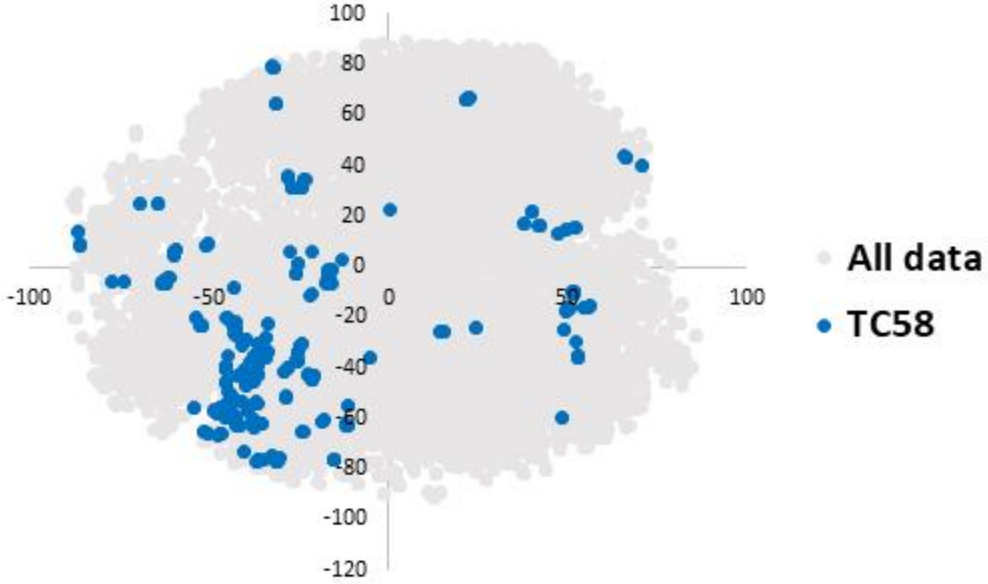

100
80
60
40
20
0
-20
-40
-60
-80
-100
-120
-100
-50
0
50
100
All data
TC58

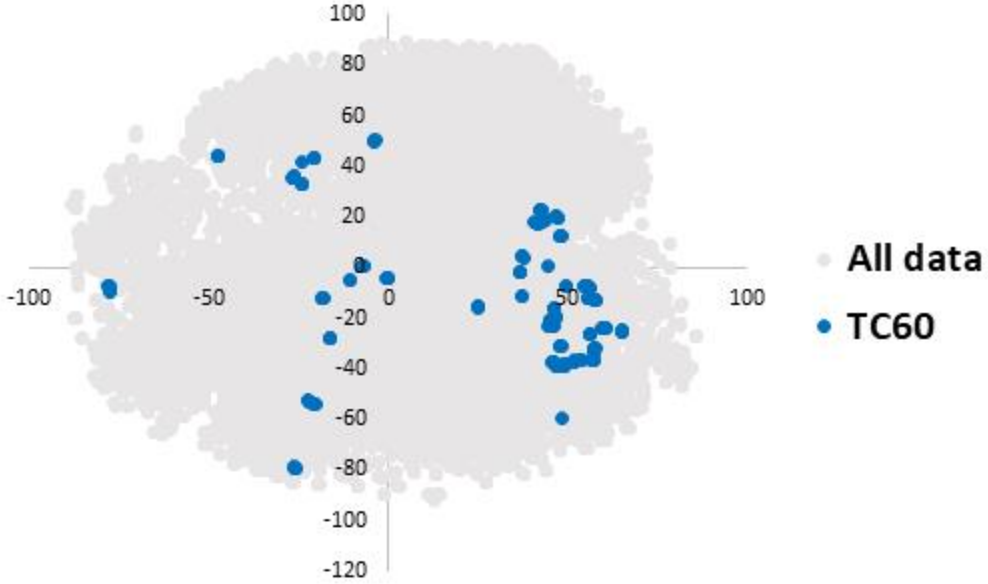

100
80
60
40
20
0
-20
-40
-60
-80
-100
-120
-100
-50
0
50
100
All data
TC60

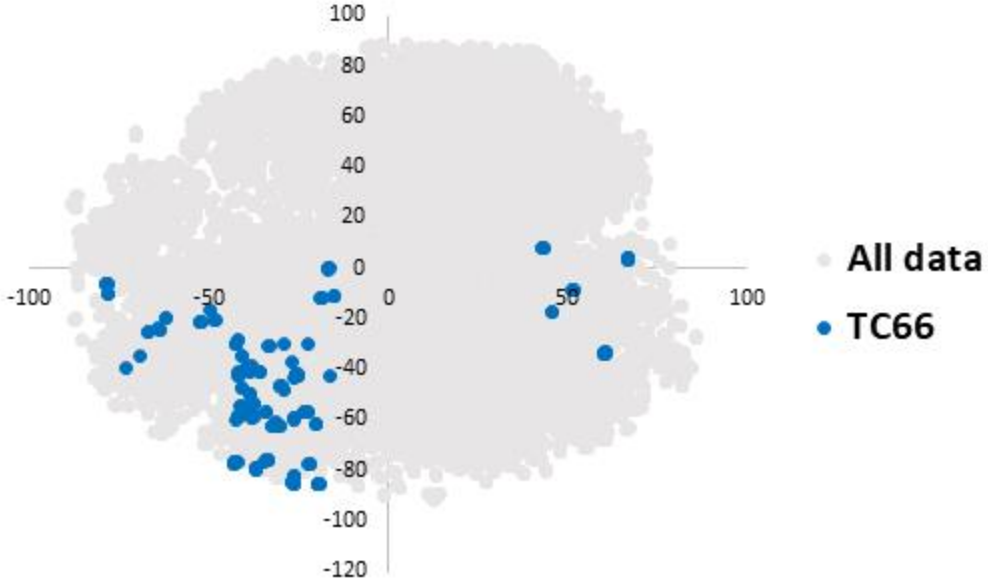

100
80
60
40
20
0
-20
-40
-60
-80
-100
-120
-100
-50
0
50
100
All data
TC66

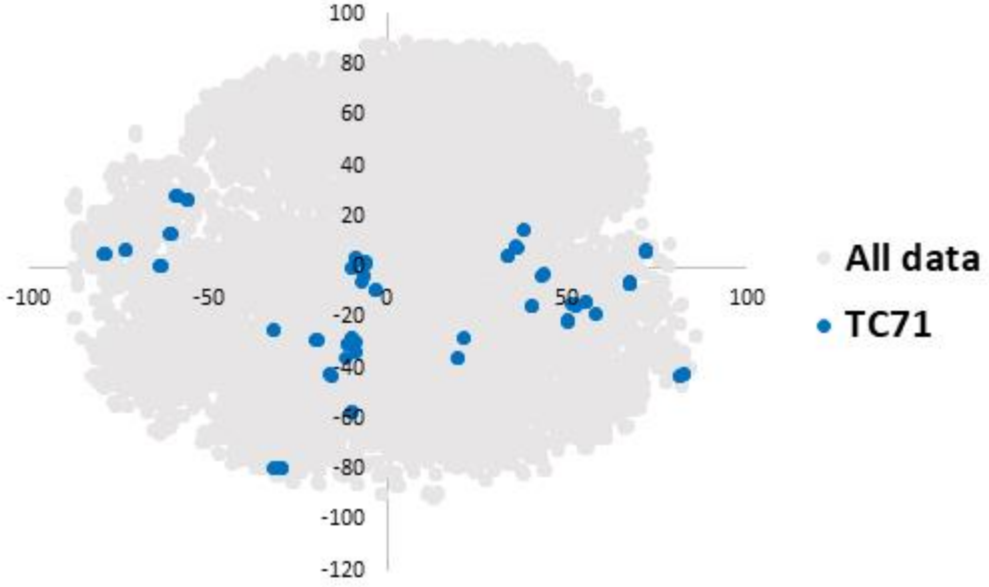

100
80
60
40
20
0
-20
-40
-60
-80
-100
-120
-100
-50
0
50
100
All data
TC71

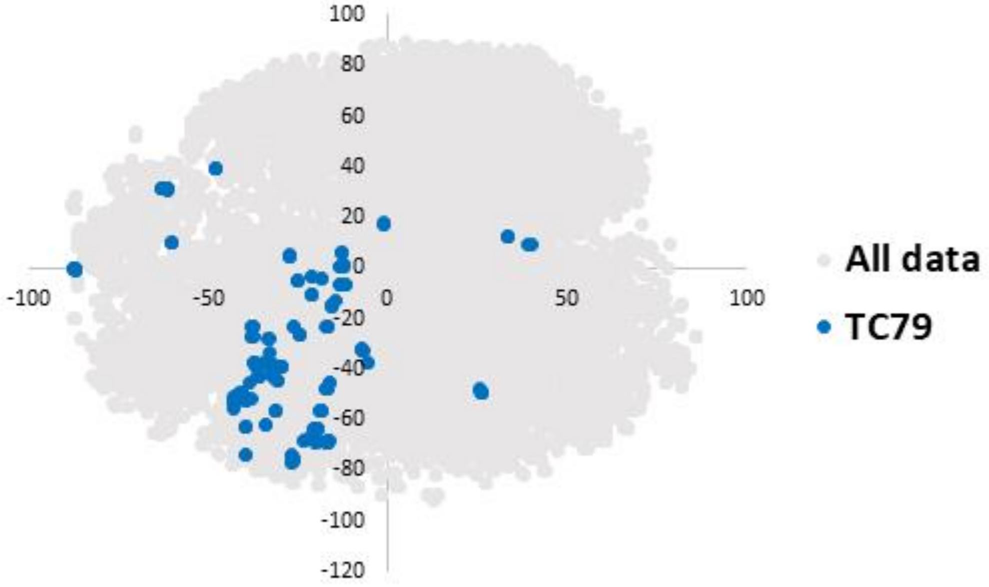

100
80
60
40
20
0
-20
-40
-60
-80
-100
-120
-100
-50
0
50
100
All data
TC79

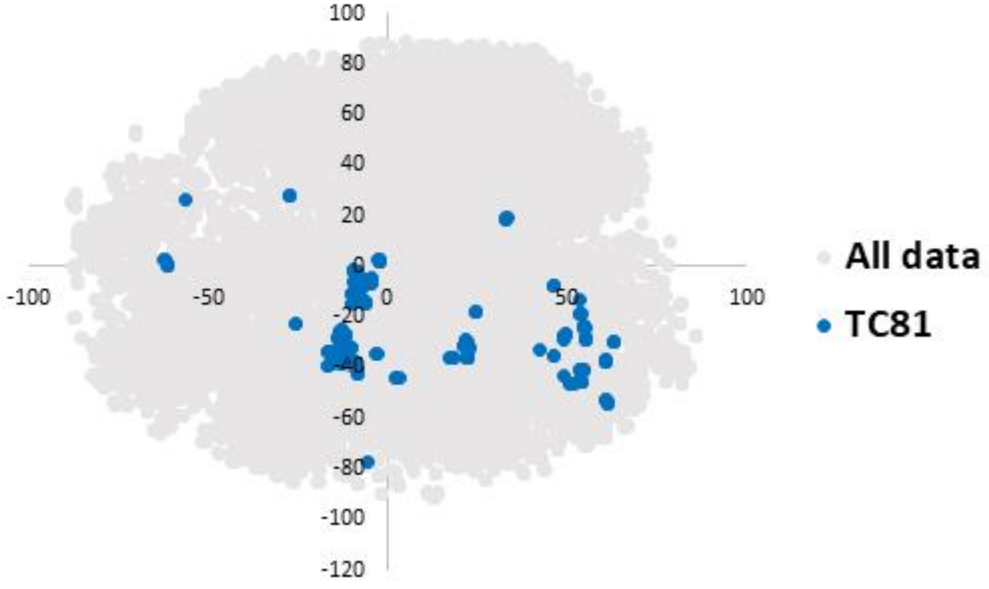

100
80
60
40
20
0
-20
-40
-60
-80
-100
-120
-100
-50
0
50
100
All data
TC81

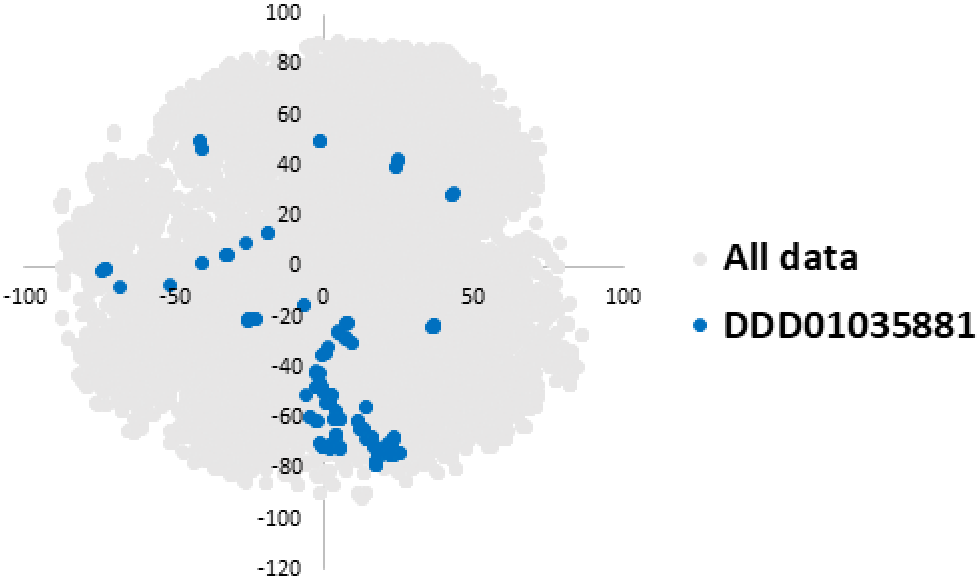
100
80
60
40
20
0
-20
-40
-60
-80
-100
-120
-100
-50
0
50
100
All data
DDD01035881

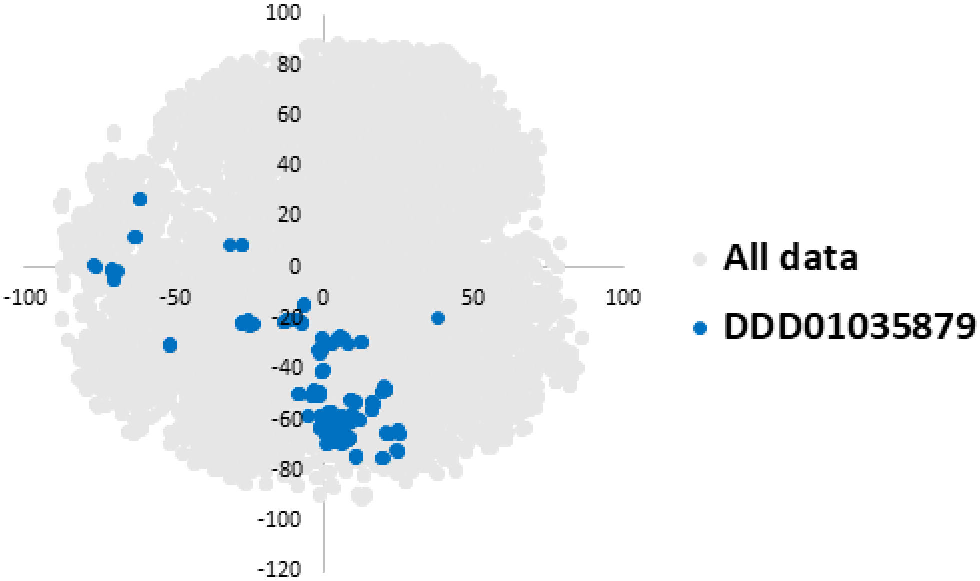
100
80
60
40
20
0
-20
-40
-60
-80
-100
-120
-100
-50
0
50
100
All data
DDD01035879

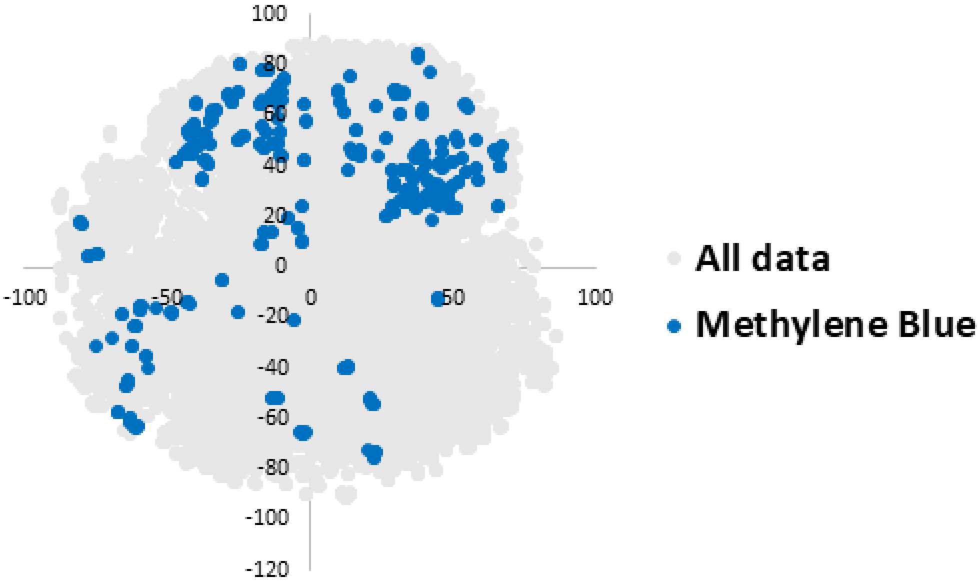
100
80
60
40
20
0
-20
-40
-60
-80
-100
-120
-100
-50
0
50
100
All data
Methylene Blue

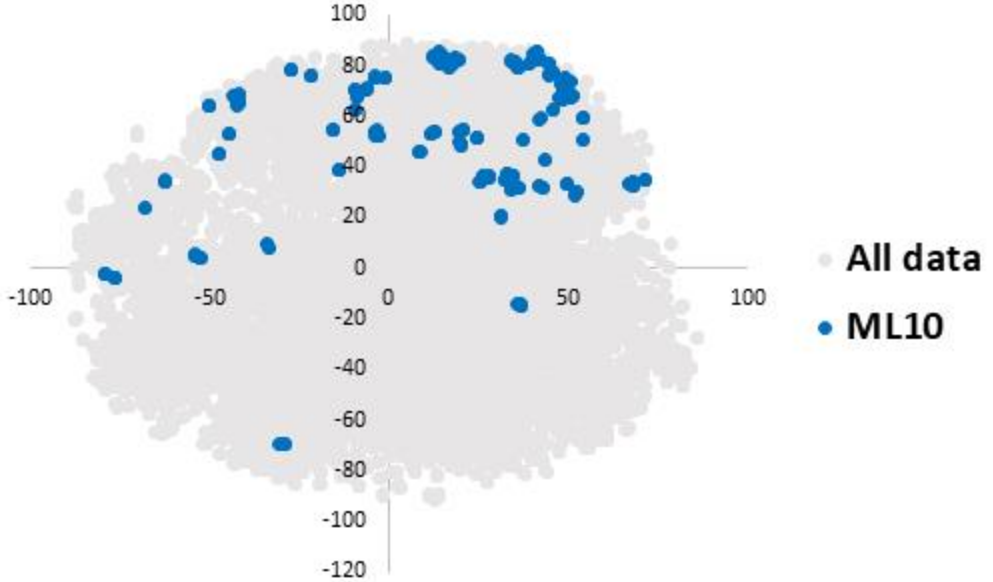

100
80
60
40
20
0
-20
-40
-60
-80
-100
-120
-100
-50
0
50
100
All data
ML10

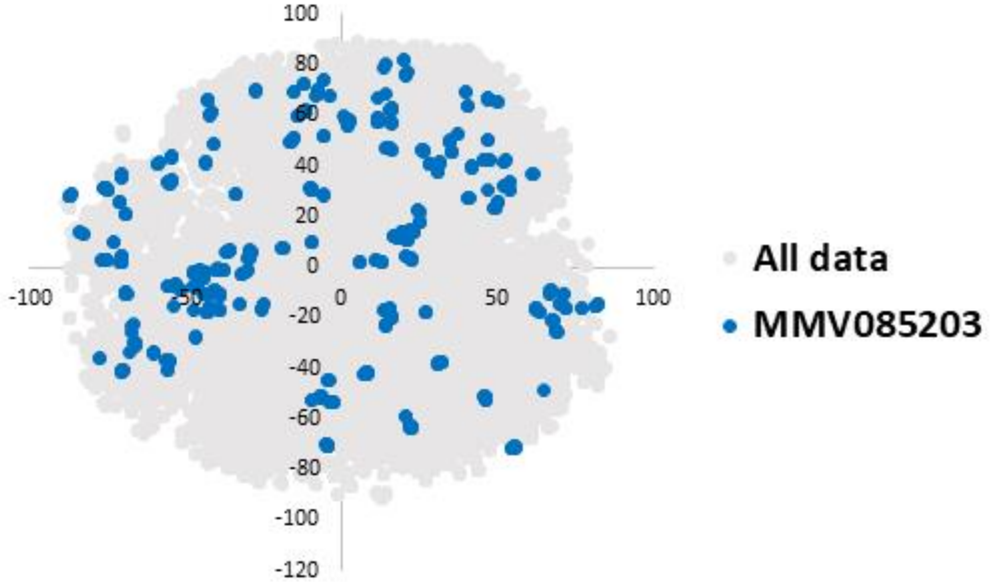

100
80
60
40
20
0
-20
-40
-60
-80
-100
-120
-100
-50
0
50
100
All data
MMV085203

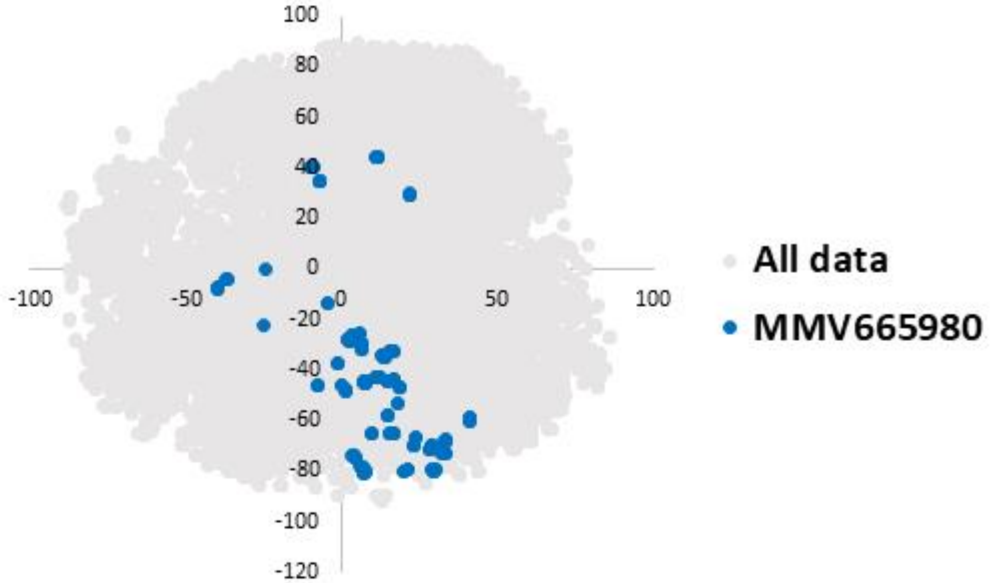

100
80
60
40
20
0
-20
-40
-60
-80
-100
-120
-100
-50
0
50
100
All data
MMV665980
