# Supplementary material for: Machine learning-based phenotypic imaging to characterise the targetable biology of Plasmodium falciparum male gametocytes for the development of transmission-blocking antimalarials
Source: PLoS Pathog. 2023 Oct 6;19(10):e1011711. doi: 10.1371/journal.ppat.1011711 (PMC10584170; doi:10.1371/journal.ppat.1011711)
Supplement: S3 Fig — The percentage of cells from each drug treatment falling into each of the 9 identified clusters was compared by PCA. All nine computed principle components were then used to cluster each drug phenotype by k-means clustering and the elbow method which determined 5 clusters was optimal. Cluster assignment is summarised in Fig 5. Bar = 3μm. (PDF) [file ppat.1011711.s006.pdf]

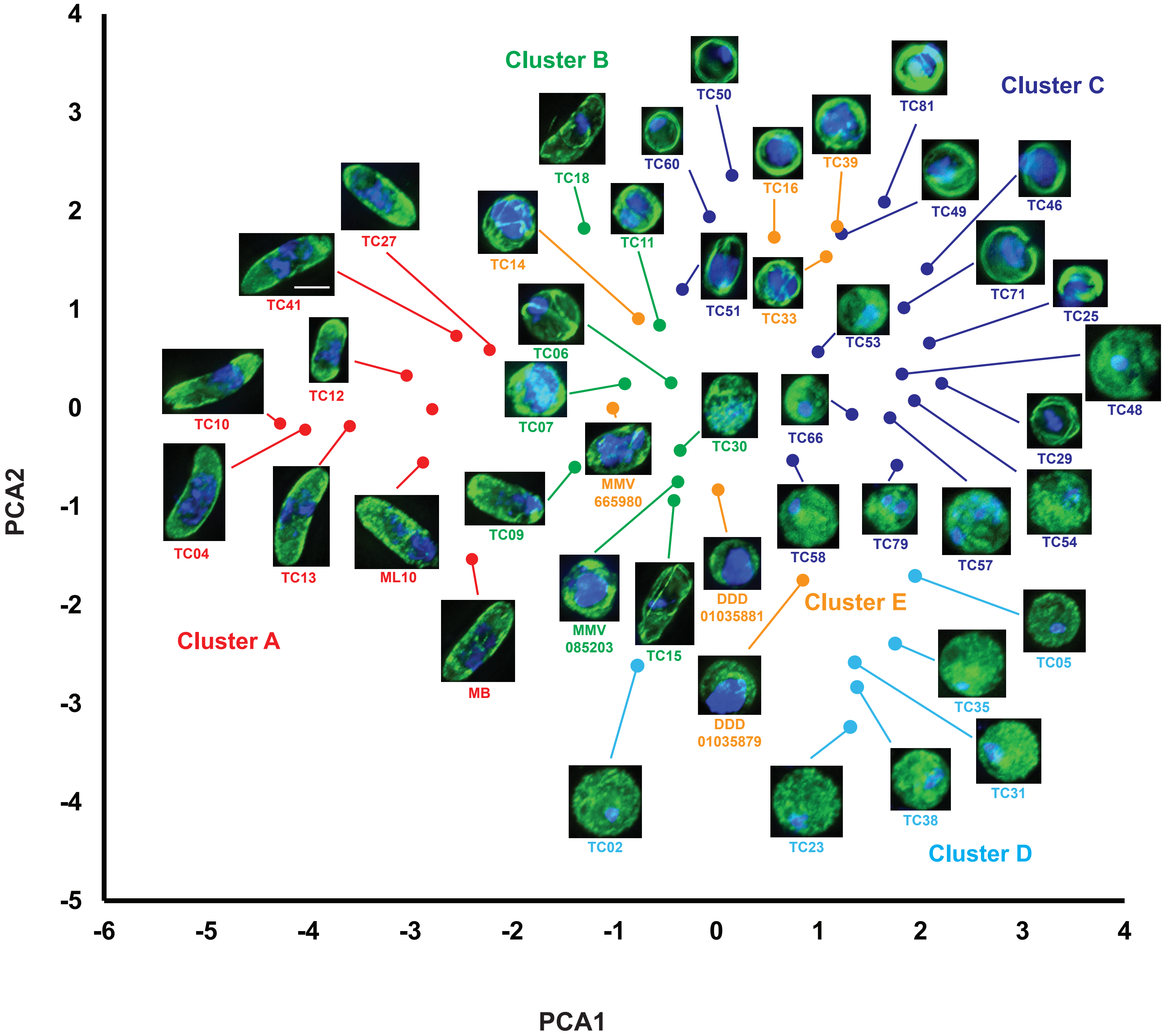

Supplementary Figure 3 - Principle component analysis (PCA) plot of the first two principle components (representing 56.8% of the total variance) of the cluster assignments from Figure 4. The percentage of cells from each drug treatment falling into each of the 9 identified clusters was compared by PCA. All nine computed principle components were then used to cluster each drug phenotype by k-means clustering and the elbow method which determined 5 clusters was optimal. Cluster assignment is summarised in Figure 5. Bar = 3μm.
