# Supplementary material for: Machine learning-based phenotypic imaging to characterise the targetable biology of Plasmodium falciparum male gametocytes for the development of transmission-blocking antimalarials
Source: PLoS Pathog. 2023 Oct 6;19(10):e1011711. doi: 10.1371/journal.ppat.1011711 (PMC10584170; doi:10.1371/journal.ppat.1011711)
Supplement: Attachment — Submitted filename: Response to reviewers v2.docx [file ppat.1011711.s007.docx]

**Response to Reviewer’s Comments**

Reviewers comments are in regular text, our responses are in **BOLD**.

**We thank both reviewers for their supportive, thorough and constructive comments. We have endeavored to answer all comments, making changes where appropriate and justifying decisions where not.** **Please find included a point-by-point response to all comments, a revised manuscript with changes indicated (all line numbers refer to this version), and a “clean” revised manuscript.**

**Of major change, as requested by Reviewer 2, we have altered some of the parameters in the analysis (augmenting images and allowing the software to consider k-means clusters in increments of 1 rather than 5). As a consequence, all data figures have needed to be replaced, however the reported outcomes are highly similar to the previous reviewed version and so the major findings and conclusions remain largely unaltered.**

**Reviewer #1:** The manuscript by Tsebriy et al. outlines and applies a custom modular pipeline based on open-source software to segment and characterise sexual stage Plasmodium falciparum parasites. Using known and uncharacterised compounds they demonstrate the workflow clusters these compounds together – based on their modes of action. They go on to show how parasite activation – particularly male gametocyte activation - is temporally sensitive to some of these drug candidates. This demonstrates the workflow can be used to define effects of compounds of gametocytogenesis through time.

This work demonstrates a timely piece of research which would be of great value to the scientific community. However, the manuscript would benefit from clarification in certain areas which are outlined next.

**Specific comments addressed below.**

**Reviewer #2:** The manuscript by Tsebriy and colleagues proposes the use of a machine learned (deep neural network) based pipeline (PhIDDLI) to classify images of P. falciparum male gametocytes/gametes treated with antimalarial compounds as a way to associate specific morphological features of treated parasites to chemical scaffolds to help elucidating compound(s) mechanisms of action.

The work is inspired by the approach described in reference 15, in which images capturing the continuum of cellular modifications occurring during the development of asexual parasites were subjected to a similar deep neural network based classification and the algorithm was then tested on parasites treated with compounds active on specific cellular targets. I have some fundamental concerns on the pipeline presented here, starting from some baseline methodology aspects (Part II of the review).

Even though morphology alterations caused by drug treatment are, as authors state, just a preliminary glimpse into modes of actions, the authors’ effort to develop artificial intelligence approaches for the desirable scaling up of transmission blocking drug discovery is indeed important.

**We thank the reviewer for recognising the importance of our work.**

However the claim that PhIDDLI is adequate to the task and that can already contribute to this goal on other systems is an overstatement.

**Respectfully, we disagree with the reviewer on this point. We state in the final sentence of the abstract: “*Our analysis pipeline is not optimised for a specific organism and could be applied to any fluorescence microscopy dataset containing cells delineated by bounding boxes, and so is extendible to any disease model.*” What we have stated is factually correct and does not overstate the role PhIDDLI could play in analysing other (similar format) datasets from other disease models. We accept that this has not been formally tested and so have modified this statement to be more cautious “*and so is potentially extendible to any disease model*” (Line 31).**

Analysis of some of the results of the pipeline presented here casts doubts on machine learning performance and suggests that visual inspection still plays an important role in this analysis. For instance, the parasite morphologies shown in Figures 6 and 7, respectively named “multiplets” and “coffee beans” do not seem to be at all represented in the PhIDDLI features of Figures 3 and 4.

**We believe that this is a misunderstanding of the data presented and apologise if there is confusion on our part. The “multiplet” and “coffee bean*” cell morphologies were identified in Figures 6 and 7 as part of the short term incubation timecourse analysis. They are not identified in the initial primary screen dataset of Figures 3 and 4 where gametocytes were incubated for a prolonged 24h period simply because they do not exist under this experimental set up (i.e. the “long incubation” phenotype of these particular molecules masks these phenotypes). As discussed in the manuscript on lines 430-433, these distinct phenotypes are transitory and only arise if gametogenesis is inhibited by the particular molecules at temporally distinct periods. In addition, it is not claimed in the manuscript that PhIDDLI removes the need for “visual inspection”. It is a tool to help analyse large image datasets in a systematic, repeatable and unbiased manner to help researchers accelerate their study of cellular phenotypes. It is still for the researcher to derive meaning from the output of the analysis.**

***Please note that in the revised manuscript reference to the coffee “bean” phenotype has been removed as under the new analysis conditions outlined below this very small population of cells was not identified as an independent cluster.**

**Part II – Major Issues: Key Experiments Required for Acceptance**
Please use this section to detail the key new experiments or modifications of existing experiments that should be absolutely required to validate study conclusions.

Generally, there should be no more than 3 such required experiments or major modifications for a "Major Revision" recommendation. If more than 3 experiments are necessary to validate the study conclusions, then you are encouraged to recommend "Reject".

**Reviewer #1:** 1. As the authors use maximum intensity projection, 3D information is lost. The authors should comment on whether this reduces sensitivity of the classification. For example, in Figure 4, the example images of Cluster 11 and Cluster 7 or Cluster 4 and Cluster 9 could easily fall into the other category.

**We agree that some information is lost through transforming 3D slices of individual cells into a single 2D image. However, *Plasmodium* parasites are very small and with the resolution achievable using deconvolved widefield microscopy (which we selected as a microscopic technique as a compromise between speed of acquisition, technology available to us, and technology most widely available to others), very little 3D information is resolvable in z-stack images. By performing maximum intensity projections, we are actually increasing the data in each cell image and also removing potential bias if similar cells were imaged at different z-planes within the sample.**

Can the authors show images belonging to specific clusters that have been manually altered (scaled, flipped, rotated) are still classified in those same clusters.

**We thank the reviewer for the excellent suggestion of augmenting the dataset. We have now added an augmentation step to our pipeline (see Fig. 3, lines 208-211 in the text) which rotates each image in 4 orientations and mirrors them. Essentially this has expanded our datasets eight-fold.** **Examining the raw data, orientation and mirroring cells does occasionally affect their cluster assignment – usually with cells that located on the border between two cluster boundaries. This effect has been reduced by altering the k-means parameters (detailed below) used to consider optimal cluster number in increments of 1 rather than 5 as previously. This has had the effect of generally reducing the optimal number of clusters calculated by the elbow method. For example, the primary dataset (Fig. 4) has reduced from 15 clusters to 9 and e.g. the number of clusters representing unactivated gametocytes has reduced from four to two.**

2. As the EfficientNet-B1 model is pre-trained for image processing, it may be missing or over-fitting certain data, can the authors comment on this. Additionally, EfficientNet-B1 achieves under 78% accuracy, while B7 now achieves 84% accuracy – can the authors run analysis non the most current model for greater robustness?

**EfficientNet are a series of models used for feature extraction and image classification. The metrics quoted by the reviewer refer to the ability of particular models to correctly identify the object in an image (i.e. ability to process a series of images and correctly identify all the images that e.g. contain a cat). In our pipeline, we are using EfficientNet for feature extraction only. Therefore the classification metrics are not applicable and using the latest version of EfficientNet does not necessarily lead to better analysis. The key difference between EfficientNet-B01 (used in our pipeline) and EfficentNet-B07 is that B07 can accept images of a higher resolution - 600x600 pixels compared to 240x240 pixels in B01. However, as *Plasmodium* cells are tiny, even with a x100 objective, individual cells in our dataset are around 240x240 pixels – the size optimal for B01. To utilise B07, images would be mostly empty space which would not add any extra information to the analysis and may actually confound it. Nevertheless, we implemented the pipeline using B07 to evaluate this. Data generated was similar to B01, however naturally, cluster arrangement was slightly different and harder to phenotypically assign:**

***Comparison of two EfficientNet models on the primary screening dataset. Blue indicates cells from the unactivated DMSO control and red indicates cells from the activated DMSO control. (left): Analysis with EfficientNet-B01; (right): Analysis with EfficientNet-B07.***

**In addition, the increase in image size accepted by B07 greatly increased processing times from 2 hours (B01) to 20 hours (B07). Therefore, we maintain our decision that EfficientNet-B01 is the appropriate model for our dataset. However, our pipeline has the flexibility to drop in any EfficientNet model to tailor the most appropriate model for image size being analysed. A brief discussion of this has been added to the text on lines 215-218.**

**Reviewer #2:** The first methodology concern is related to the nature and the number of the fluorescent markers used to stain cellular structures. Use of only two probes, i.e. DAPI for parasite nuclear staining and an antibody against tubulin (on which further comments are below), fails to reveal additional important features which could be harnessed for classification such as mitochondrial and erythrocyte surface staining. In fact, visual inspection of the features obtained before and after dimensionality reduction (Figure 3) easily spots virtually indistinguishable morphological patterns.

**We completely agree that increasing the number of cellular markers will increase the breadth of cell biology studied in an analysis. Indeed we make exactly this point in the second paragraph of the discussion - suggesting similar markers to the Reviewer for future work (intended to be performed subject to future funding applications). Our manuscript reports our initial data with two very powerful markers that encompass two major cellular events during male gametogenesis – DNA (DNA replication and reorganization), and microtubule structures (shape change, endomitosis, spindle pole formation, flagellum organization). These two markers alone already powerfully distinguish the activity of the transmission-blocking molecules into activity groups for further characterization. Rather than performing a screen with additional markers simultaneously which could increase the technical complexity of the assays and analyses, we favour targeted follow up based upon the initial classification in our DNA/tubulin screen. For example, mitochondrial inhibitors appear to arrest male gametogenesis late in the process, therefore it would be more efficient to focus on our “late” cluster of inhibitors with a mitotracker readout.**

The second concern is that no mention is present in the manuscript of how PhIDDLI deals with the fact that the majority of parasites imaged in the control and treated wells are female gametocytes/gametes. In the NF54 parasite line used here the gametocyte sex ratio (proportion of males) is strongly female biased, around 1:8 1:10. In this respect, whilst the rationale of labelling microtubules appears logical, as indeed these structures are assembled only in male gametogenesis, the anti-alpha tubulin antibody used here is not gametocyte sex specific. In fact, the statement that the antibody is against alpha-tubulin II (section 2, Results) is incorrect, as the reagent is a monoclonal antibody labelling tubulin across several mammalian species (Materials and Methods), and ambiguous, as it is reminiscent of the P. falciparum protein tubulin alpha II. Antibodies against the latter, nevertheless, are not male gametocyte specific, but show slightly different staining patterns in the cytoplasm of female and male late gametocytes ([https://pubmed.ncbi.nlm.nih.gov/21209927/](https://protect-eu.mimecast.com/s/5qKcCmy54uvWQ6PIB-yrA?domain=pubmed.ncbi.nlm.nih.gov)).

**We agree with the reviewer that the antibody used was raised against chicken embryo brain alpha-tubulin and so cannot be considered “specific” for *P. falciparum* alpha-tubulin II (though is routinely used by many *Plasmodium* researchers). Whilst the MM section does not claim this and specifically states the monoclonal used so that our data can be reproduced, we have clarified this line 625.**

**We are well aware of the article cited by the reviewer which was published by colleagues here at LSHTM. It reports that female gametocytes express indistinguishable levels of tubulin than males by IFA, but crucially, only when immature. It is important to note, our study only focuses on mature (Stage V) gametocytes with the ability to undergo gametogenesis. The cited paper states that mature females show much fainter anti-tubulin immunoreactivity which is even more apparent after gametogenesis is induced [copied directly from their paper, passages underlined for emphasis, Pfg377 is a specific female gametocyte marker]:**

**“*From stage I to III, α -tubulin II fluorescent intensity was similar among all gametocytes at the same stage of maturity, yet stage IV and stage V gametocytes that reacted with anti-Pfg377 revealed much lower expression levels of α-tubulin II than those gametocytes not expressing Pfg377 (Fig. 4C, D, E). Particularly striking was the strong fluorescence in activated gametocytes that only reacted with anti-α-tubulin II antibodies and not Pfg377 antibodies (presumably male), whereas the activated gametocytes also reacting with Pfg377 (presumably female) showed very low expression levels of α-tubulin II (Fig. 4E).*”**

**The semi-automated cell detection in ICY Bioimage Analysis filters out cells faintly reactive for alpha-tubulin (line 639). Therefore we are confident that our dataset contains predominantly male gametocytes/gametes.**

The third concern is that I would have expected that a baseline step of the machine learning approach was dedicated to classify the many steps of a normal, unperturbed male gametogenesis, that authors rightly describe to occur in vitro as “an inefficient process that not every male gametocyte completes after stimulation”. Introduction of a training set of images encompassing the events of a normal, yet inefficient male gametogenesis, would likely help to specifically identify and classify alteration in parasite morphology induced by compounds.

**We thank the reviewer for this suggestion and indeed this would be an appropriate approach if we were monitoring the process of male gametogenesis over time. However, the approach we have taken here is “endpoint analysis”. We have triggered male gametogenesis under a variety of drug treatments or conditions and then fixed all of the cells at the “endpoint” (20 mins after triggering gametogenesis) regardless of whether they reached exflagellation or not. Under these conditions, frequently cells arrest before exflagellation (or do not commence at all). This does not mean that in our experiment we have cells representing snapshots of “normal” gametogenesis over time. Rather, we have a proportion of untreated cells that for whatever reason stalled and likely continued on aberrant development for the rest of the experiment. In this case, our control conditions added to every experiment were gametocytes that were not triggered to undergo gametogenesis (i.e. representing the most severe phenotype we could expect to observe) and exflagellating male gametes (i.e. representing a phenotype present in untreated cells). Therefore, generating such a dataset as the reviewer describes, whilst possible, would not help us with our goal of studying the phenotype of the identified transmission-blocking molecules. To better convey this point, Figure 4 has been updated to include an additional graph (Fig. 4A) that maps the controls to the overall tSNE distribution. As can be observed, the untreated cells form a focal point in the centre which represents the cells that progressed “correctly” all the way to exflagellation, but also are scattered in the lower right quadrant representing cells that arrested “late” but before exflagellation. In addition, for further transparency/clarity, Supplementary Figure 2 has been created which maps the distribution of each drug to the overall t-SNE distribution. From this it is clear to see that different drugs do drastically alter distribution of the cells.**

**Part III – Minor Issues: Editorial and Data Presentation Modifications**
Please use this section for editorial suggestions as well as relatively minor modifications of existing data that would enhance clarity.

Reviewer #1: Additional issues, that are less impactful to the integrity of the work but should nonetheless be addressed include:

3. Page 6: Clarity is needed on how efficient the acquisition to analysis is. ‘Images (n = 1207) and bounding boxes… and extraction of individual cells (n = 2446)’. Does this mean only 2 cells were extracted from each captured image?

**At the effective x150 magnification we imaged our cells at, and at the cell densities we seeded to prevent overcrowding, we observed a variety of cells in each acquired image (1 to ~8). The manuscript has been updated to indicate this (Line 642).**

4. Scale bars should be added to Figures containing images.

**Apologies for the oversight, these have now been added.**

5. Images were extracted for ‘Tubulin-bright cells’, what if the drug ablated microtubules completely? This data would be lost, can the authors comment on this?

**To the best of our knowledge we are not aware of a drug that ablates tubulin expression entirely. If such a drug exists then definitely it would be lost from our analysis. However, as illustrated in Figure 6, treatment with the microtubule depolymerizer vinblastine still gives readily detectable cells and a distinct phenotype.**

6. Figure 3: Please make axis labels in any final versions readable.

**The figures in the initial submission were converted to BMP format. The revised version also includes high resolution versions of all figures to ensure legibility.**

7. Figure 6 title: remove ‘the’: ‘Comparing the how male…’

**Removed**

8. Figure 7: It seems the ‘Coffee bean’ phenotype (Pink cluster) is distributed in both the extreme positive and negative of the x axis. Why are they separated so widely?

**Using the new analysis parameters, the small clusters possessing the “coffee bean” phenotype have been absorbed into other clusters due to their very minor representation. To avoid “cherry picking” data, as the new analysis does not identify these as a distinct cluster (we suspect because there were so few cells), we have removed reference to it in the text.**

9. Page 12: 1st paragraph, shorten the last sentence which is 5 lines in length.
**This sentence has been split in two.**

10. Page 12: 2nd paragraph, Clarify which Figure they are referring to when citing ‘(Cluster A)’
**Corrected (line 476)**

11. Page 12: Final section before Discussion – Making the summary of compounds into a table would make this much easier to navigate.

**Figure 8 has been created to summarise the activity of the studied compounds.**

Reviewer #2: With the above fundamental concerns, minor questions on details of the pipeline are
1- the reason for having 1000 features extracted by the staring set of 2446 cells from the 1027 images;

**The EfficientNet-B01 model extracts 1000 features from each cell passed to it rather than 1000 features across the entire dataset. This is inherent to the design of the model and not a user defined parameter that can be changed. 1000 features will be extracted from each cell image regardless of the number of images passed to the model. This is clarified on line 215.**

2- the parameters or rationale leading to set to 15 the number of clusters in the k-cluster analysis means;

**To save computing time, our original pipeline only evaluated clusters in increments of 5. This apparently lead to 15 clusters being always computed as optimal by the elbow method. We have revised this so that now the pipeline evaluates clusters in increments of 1. This has allowed a more sensitive calculation of optimum cluster number with different datasets yielding different cluster sizes – the primary screen now possesses 9 clusters; the timecourse assays now possess 7-11 clusters.**

3- what is the percent of variance associated to the PCA components plotted in Fig. 5;

**The PCA plot in Fig. 5 (now moved to Supplementary Figure 3) shows 56.8% variance in the first two principle components. We recognise that this is low and may confuse the reader as the intention is to demonstrate the computed cluster assignments. The cluster assignments from the PCA analysis are calculated using all PCA components (100% of the variance). Therefore for clarity, we have replaced Fig. 5 with a modified version showing images of exemplar cells from each drug treatment ordered by the cluster they are assigned to. We feel that this is easier for the reader to understand and better illustrates the power of our analysis.**

4- what images were used to pre-train the EffcientNet-B1 module, the open source deep neural network of PhIDDLI.

**EffcientNet was trained on the open ImageNet image dataset (**[**https://www.image-net.org/**](https://www.image-net.org/)**) of >14 million images with ~20k different classifications (such as “cat”, “dog”, “car” etc). We use the model “off-the-shelf” without any additional training on images of cells or a sample of our dataset. This is possible as we are not using it to classify our cells, we simply use the module to extract numerical features from our cells which are then used to cluster cells with similar phenotypes using k-means clustering and visualized by the the dimensional reduction tool tSNE.**
